# From Seaweed to Science: Alginate Scaffolds in Shaping the Future of Tissue Engineering

**DOI:** 10.3390/molecules31213957

**Published:** 2026-11-02

**Authors:** Koushik Saha, Konkona Belel, Santanu Singh, Shounak Sarkhel, Ranu Biswas, Delia Mihaela Rata, Leonard Ionut Atanase

**Affiliations:** 1 Department of Pharmaceutical Technology, Jadavpur University, Kolkata 700032, WB, India; saha39594@gmail.com (K.S.); konkonabelel@gmail.com (K.B.); santanusingh151@gmail.com (S.S.); 2 Department of Pharmaceutical Technology, JIS University, Agarpara, Kolkata 700109, WB, India; shounak2013@gmail.com; 3 Faculty of Medicine, Apollonia University of Iasi, 700055 Iasi, Romania; iureadeliamihaela@yahoo.com; 4 Academy of Romanian Scientists, 050045 Bucharest, Romania

**Keywords:** sodium alginate, scaffolds, tissue engineering, hydrogels, biomedical applications

## Abstract

Alginate, a natural polysaccharide primarily derived from brown seaweed, is a promising biomaterial for tissue engineering because of its biocompatibility, mild gelation conditions, and tunable physicochemical properties. Alginate-based scaffolds can support cell encapsulation and the localized delivery of therapeutic agents, making them suitable for diverse regenerative applications. Recent advances in crosslinking strategies, chemical modification, nanocomposite incorporation, and fabrication techniques such as three-dimensional (3D) bioprinting have improved scaffold stability, bioactivity, and functional performance. These developments have expanded the potential applications of alginate-based scaffolds in bone, cartilage, skin, neural, and cardiac tissue engineering. However, limitations associated with intrinsic cell adhesion, mechanical strength, and degradation control remain important challenges. Although preclinical studies have demonstrated promising results in several tissue-engineering applications, further investigation is needed to establish long-term functional outcomes, safety, and clinical efficacy. This review discusses the evolving role of alginate scaffolds, from marine-derived hydrogels to engineered biomaterial platforms, and highlights their potential and remaining challenges in next-generation tissue regeneration.

## 1. Introduction

From the evolutionary era to the dynamic life of current times, a wide range of activities have been implemented, leading to a great deal of human contact and exchange, which will soon improve conditions for living and advance science to previously unheard-of levels as a crucial component of global development [1,2]. The goal of biomedical engineering and regenerative medicine is to use diverse sciences in contemporary society to create a customised therapy for replacing or repairing lost or damaged organs and tissues [3]. Biomedical engineering’s regeneration strategy places an important focus on the potential to create a biomimetic 3D cellular microenvironment (artificial extracellular matrix (ECM) or scaffold) that regulates and guides local tissue regeneration. The features of nanofibers, such as their natural extracellular matrix (ECM) structure, high water absorption, high interconnected porosity, breathability, and moisture permeability, create an environment that is appropriate for blood circulation, infection prevention, cell migration and proliferation, respiration, and exudate absorption [4]. Tissue engineering with three-dimensional biomaterials represents a promising approach for developing various functional tissues to replace the function of a failing liver, cardiac, blood vessel, and bone and nerve tissue. Herein, we address cell seeding and distribution within porous alginate scaffolds, which represent a new type of porous biomaterial for tissue engineering [5]. Graphene derivative possesses unique physical, chemical, and electrical properties that can promote stem cell behaviour and enhance tissue repair [6].

Scaffolds are three-dimensional porous skeletal structures of biofibers that, depending on the type of application, contain a variety of natural or synthetic polymers that should have particular physicochemical properties (hydrophilicity, porosity, morphology, mechanical properties, biodegradability, and biocompatibility) to as closely resemble the cell environment in vivo as possible [7]. For the controlled release of therapeutic bioactive compounds (growth factors, vitamins, hormones, and antibiotics) that aid in tissue regeneration and advance the healing process, scaffolds are intended as drug delivery platforms [8]. Physicochemical characteristics like hydrophilicity, porosity, morphology, and mechanical properties can be tailored to specific applications through crosslinking, blending, copolymerization, and functionalization. Biodegradable scaffolds are made from biological sources and degrade completely after tissue regeneration [9]. Scaffold-free approaches using cell aggregates (spheroids) can also be used for tissue regeneration. The role of a scaffold is to provide shape and texture to the protein source, which can be cultivated cells or plant proteins. These agents protect and stimulate fibroblasts and adult stem cells, promoting their migration into damaged areas and facilitating new tissue formation [10].

## 2. Molecular Structure and Material Science of Alginate

**Figure 1:**
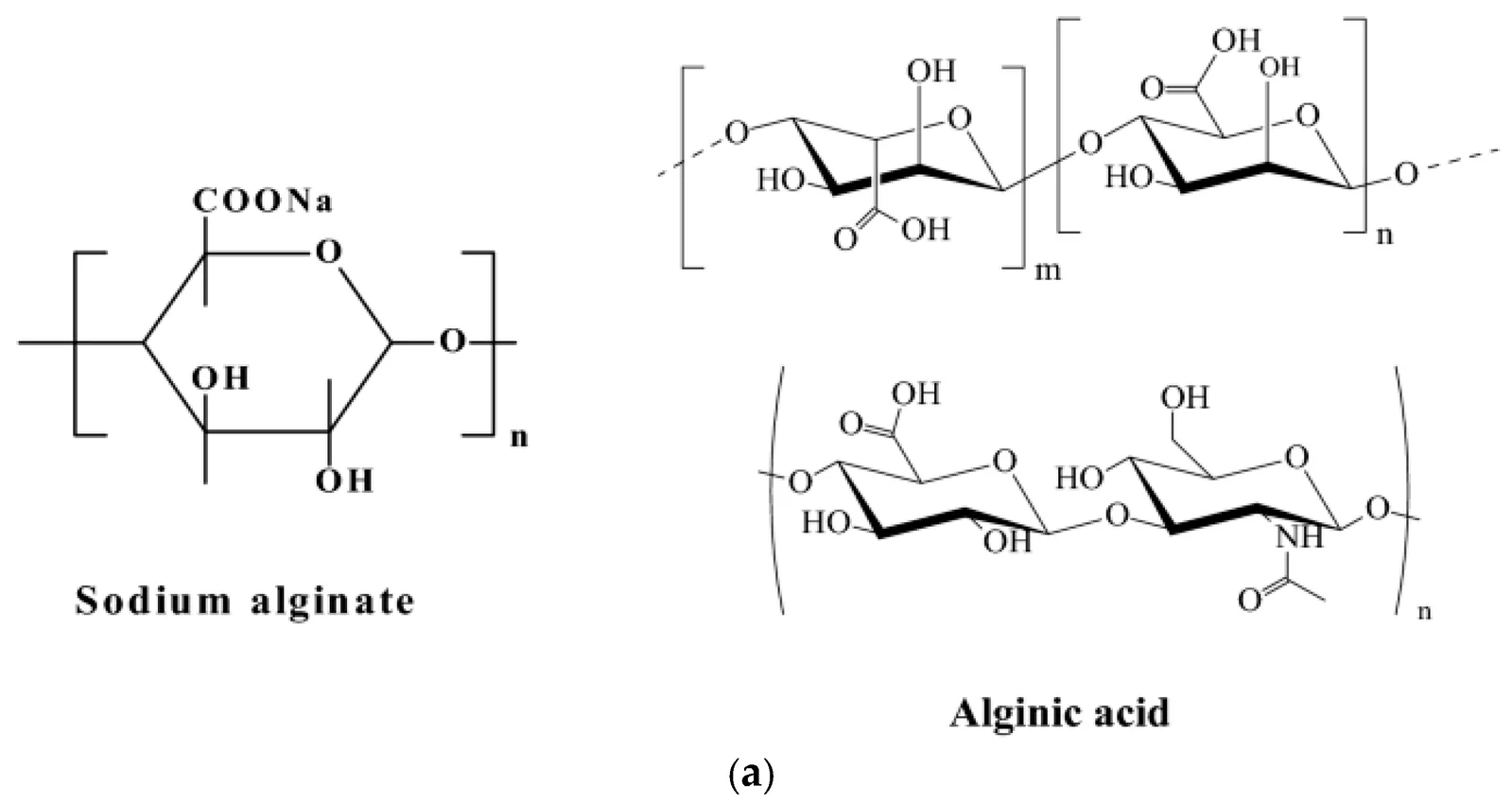

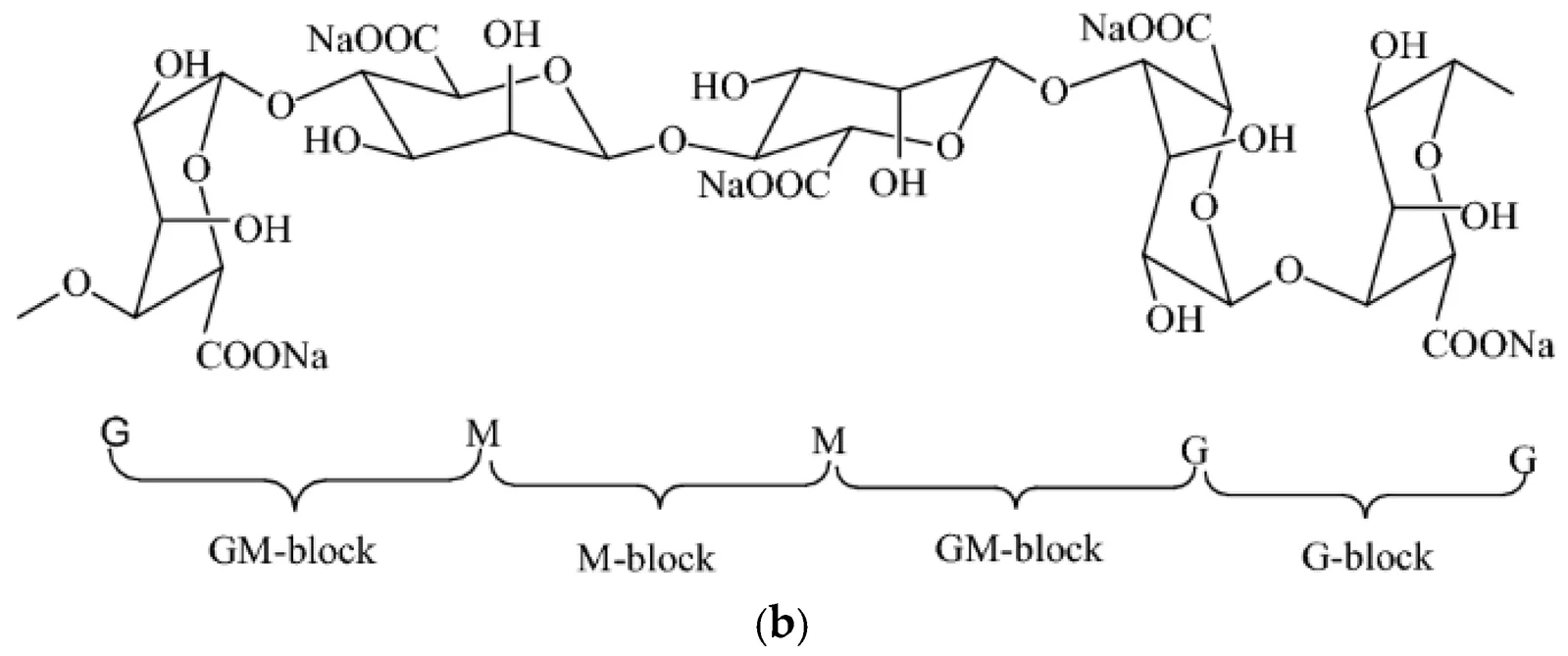
(**a**) Structure of alginic acid and sodium alginate and (**b**) conformation of monomers and block distribution of alginate [11].

Alginate is one of the most abundant natural biopolymers, after cellulose, primarily derived from marine brown algae and bacterial sources such as *Pseudomonas aeruginosa* and *Azotobacter vinelandii*. Chemically, it is a linear anionic polysaccharide composed of (1–4)-linked β-D-mannuronic acid (M) and α-L-guluronic acid (G) residues arranged in homopolymeric (M or G) and heteropolymeric (MG) blocks (Figure 1).

**Figure 2:**
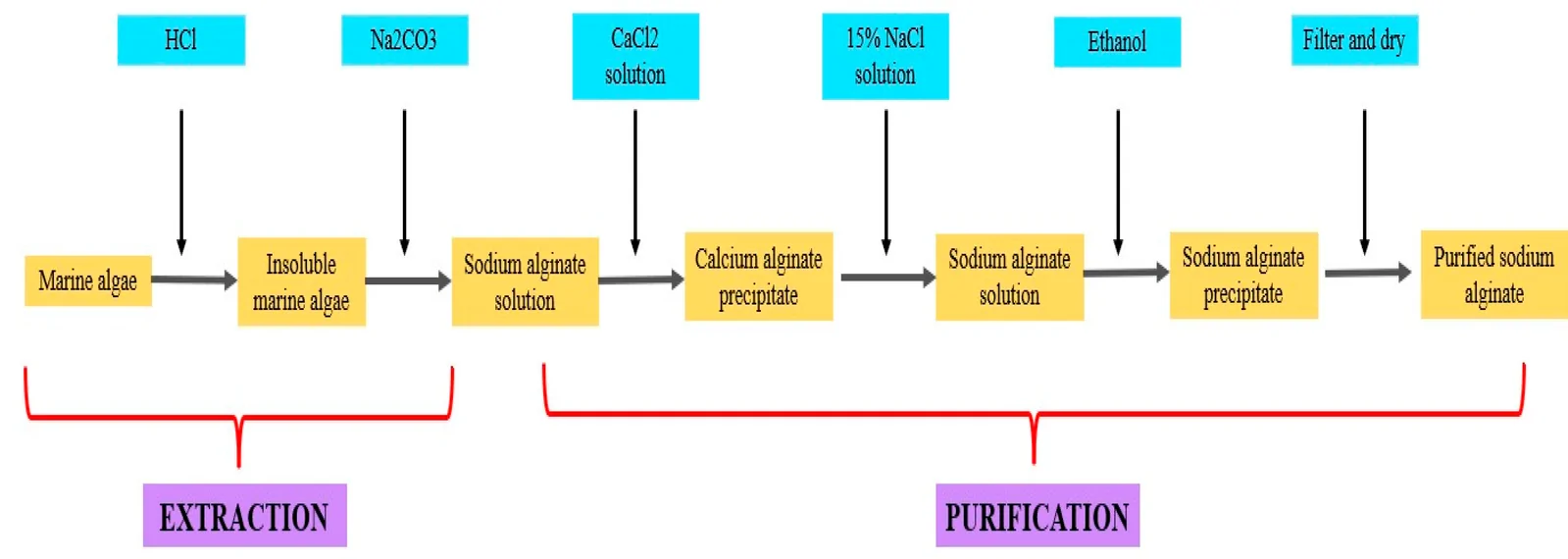
Commercial method of calcium precipitation-based sodium alginate extraction.

The M/G ratio and block length critically influence the gelation behavior, viscosity, and mechanical properties of alginate hydrogels. G-rich alginates generally exhibit stronger interactions with divalent cations, particularly Ca^2+^, than M-rich alginates, potentially resulting in stiffer hydrogel networks under comparable preparation conditions. However, the resulting mechanical properties depend not only on the M/G ratio and block distribution but also on molecular weight, polymer concentration, crosslinker identity and concentration, and experimental conditions. Although Ca^2+^ and Ba^2+^ can form alginate networks with different crosslinking strengths, Mg^2+^ generally exhibits weaker interactions with alginate and may produce less robust gels under comparable conditions. Therefore, the structure–property relationships of alginate hydrogels should be interpreted in the context of their specific composition and preparation conditions [12,13,14,15]. Industrial extraction from brown algae involves sequential pre-treatment, acid washing, and alkaline solubilization, typically using dilute HCl for ion removal followed by Na_2_CO_3_ treatment to obtain sodium alginate (Figure 2). Precipitation using ethanol, CaCl_2_, or HCl yields alginate salts or alginic acid, which is subsequently purified and dried. Algal alginates are non-acetylated and require chemical modification to achieve desired physicochemical properties, while bacterial alginates naturally exhibit variable acetylation at O-2/O-3 positions, enhancing solubility, viscoelasticity, and water retention capabilities. Azotobacter’s regulated biosynthesis allows for the creation of customised alginates by modifying fermentation conditions including pH, temperature, and nutrient content. Alginate-based materials have attracted considerable interest in wound healing, drug delivery, cell encapsulation, and tissue regeneration because of their generally favorable biocompatibility, potential biodegradability under appropriate conditions, and structural suitability for biomedical applications [16,17,18,19,20]. However, their biological performance depends on material purity, chemical modification, crosslinking chemistry, and the surrounding biological environment.

The hydrogel form of alginate enables efficient nutrient diffusion and cellular proliferation, supporting its use as a scaffold in regenerative medicine and as a carrier for site-specific drug release. Moreover, the mild gelation via ionic crosslinking with divalent cations under physiological conditions makes alginate an ideal matrix for diverse biomedical applications. In addition to the rigid six-membered sugar rings’ greater or lesser hindrance, the restricted rotation around the glycosidic bonds, and the electrostatic repulsion between the charged groups on the polymer chain, the linkage in the block structure causes alginates to vary in degree of stiffness or flexibility [21]. The stiffness and flexibility of alginate chains are influenced by their block composition and molecular structure [21]. G-rich alginates generally exhibit greater stiffness and stronger interactions with divalent cations than M-rich alginates under comparable conditions. However, the mechanical properties of alginate hydrogels also depend on molecular weight, polymer concentration, crosslinker identity and concentration, and the conditions used for gel preparation and mechanical testing. Therefore, the relative stiffness of alginate-based materials should be interpreted in the context of their composition and experimental conditions [22]. Alginic acid is insoluble in water and organic solvents, whereas its monovalent salts and esters are water-soluble and form a stable, viscous solution. The molecular weight, rigidity, polymer chain extension, and ionic strength of the solution all affect the inherent viscosity of alginates. The viscosity increases due to an increase in the molecular weight, concentration of alginate, or stiffness of the individual chain blocks. In addition, as the polymer chain lengthens, the inherent viscosity in cartilage tissue engineering rises due to the electrostatic repulsion between the charged groups on the chain [23].

## 3. Foundational Concepts Underpinning Alginate Scaffolds in Tissue Regeneration

Alginate scaffolds represent a suitable option for tissue engineering due to their biocompatibility, rapid and cytocompatibility ionic gelation, and ease of chemical modification. These properties enable the creation of cell-instructive architectures with customizable mechanics, degradation rates, and bioactivity tailored to specific target tissues. Recent reviews highlight that alginate’s shear-thinning properties and rapid Ca^2+^-mediated gelation facilitate cell encapsulation, interlayer adhesion during bioprinting, and early structural integrity [24,25]. Additionally, composite designs and ligand functionalization address inherent limitations in cell adhesion and mechanotransduction. Chemical modifications, specifically oxidation to alginate dialdehyde and methacrylate, facilitate the formation of dynamic covalent or photo-crosslinked networks [26,27,28]. These enhancements lead to improved printability, self-healing properties, and redox-responsive remodelling, thereby advancing the precise deposition and maturation of 3D constructs in the fields of cartilage, bone, vascular, and neural regeneration [29,30,31]. Advanced strategies integrate alginate with collagen, gelatin, hyaluronic acid, or chitosan, and utilize bioactive glass and nanoparticles to enhance mechanical strength, regulate ion release for in situ gelation, and provide cell-adhesive ligands that improve adhesion, proliferation, and lineage-specific differentiation, thereby mitigating the inherent limitations of pure alginate, such as low adhesion and brittleness [32,33,34]. In bioprinting, the use of “smart” and 4D alginate inks with programmable shape-morphing enhances the spatiotemporal control of scaffold geometry and microenvironment [1]. Additionally, multimodal crosslinking, which combines ionic and covalent or dynamic methods, separates printability from long-term stability, thereby improving fidelity and functional integration in vivo [35,36,37]. Recent findings indicate that alginate-based scaffolds promote the formation of vascular networks and facilitate the sustained release of morphogens or genes, which are essential for cell survival and structured tissue regeneration, especially in avascular or hypoxic environments. Recent literature collectively identifies alginate scaffolds as a modular and clinically relevant platform, where their physicochemical adaptability and compatibility with biofabrication correspond with translational priorities, including reproducible manufacturing, cell viability, hierarchical structure, and cues for integration and remodelling in complex tissues [38,39,40].

## 4. Fabrication Techniques of Alginate Scaffolds

**Figure 3:**
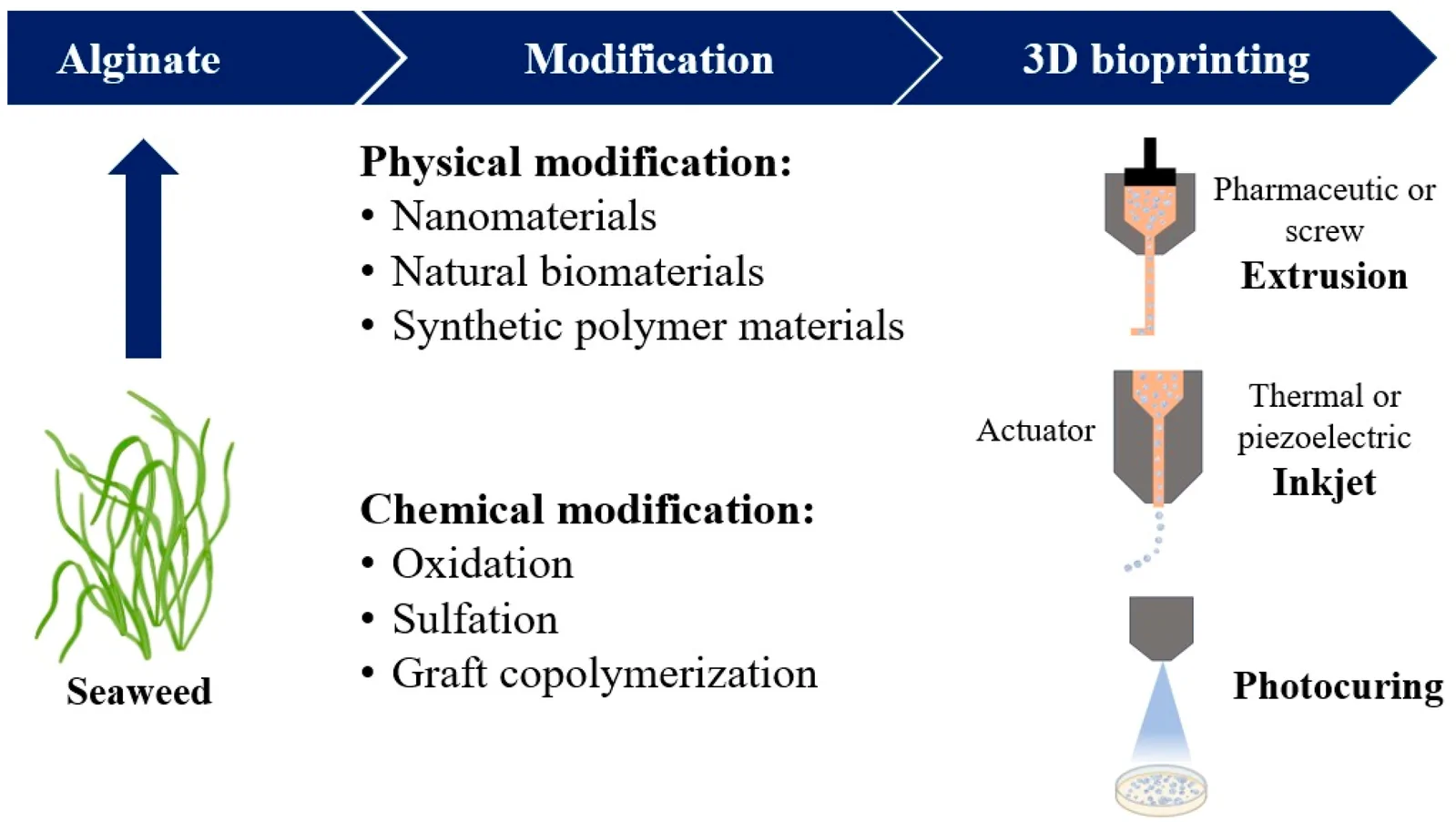
Schematic representation of alginate modification, three-dimensional (3D) printing, and tissue-engineering applications. Created based on information from relevant published studies [18].

Alginate-based biomaterials are made using a variety of methods to create hydrogels, foams, sponges, fibres, microspheres, and microcapsules. Alginate hydrogels are very desirable for a variety of uses, particularly as the delivery method or supporting matrix for tissue regeneration and repair [13]. Alginate hydrogels may be made using a variety of physicochemical crosslinking techniques that rely heavily on the sorts of components (Figure 3) [18].

### 4.1. Hydrogel Scaffolds

Hydrogels are polymeric network where water-loving polymers are crosslinked in 3D form that can swell in water by expansion of the chains and imbibe or hold a large amount of water without dissolving in [41]. Although alginate is widely used in tissue engineering because of its biocompatibility and tunable physicochemical properties, unmodified alginate lacks intrinsic cell-adhesive motifs required for effective integrin-mediated cell attachment. Consequently, cells may exhibit limited adhesion and spreading on unmodified alginate matrices. To overcome this limitation, alginate can be functionalized with cell-adhesive peptides, such as arginine–glycine–aspartic acid (RGD), or combined with extracellular matrix-derived components, including gelatin and collagen. These modifications can provide adhesive cues that facilitate cell attachment and spreading. Therefore, the cellular response to alginate-based scaffolds depends not only on alginate itself but also on its functionalization, composition, and structural characteristics [42]. It has various applications in biomedical fields but hydrogel as a scaffold form is used very popularly. Alginate hydrogels serve as excellent candidates for tissue engineering and regenerative medicine and can be easily made by crosslinking with divalent cations [43].

#### 4.1.1. Physical Crosslinking Methods

Physical crosslinking is the widely used method for the fabrication of physical hydrogel scaffolds. Dissolution of this type of hydrogels can be modulated as a function of pH or temperature [44].

##### Ionic Crosslinking (Divalent Cation)

A large number of carboxyl groups, including alginate chelate, with divalent cation were used to develop an alginate hydrogel with an “egg-box” model, which was prepared in an aqueous solution under mild conditions [45,46]. The most widely used divalent cation is calcium. Numerous studies have reported that when Ca^2+^ is used as an ionic agent for the preparation of alginate hydrogel, there is a chance of the disintegration of formed gel in a physiological environment. In this context, strontium (Sr), which is physically, chemically, and structurally similar to calcium, has become another important choice to form alginate hydrogels. Strontium also havs minimal health risk as it contains naturally stable isotopes [47,48]. Calcium ions (Ca^2+^) are widely used for ionic crosslinking of alginate because they coordinate with carboxylate groups, particularly within guluronate-rich sequences, to form three-dimensional networks commonly described by the egg-box model. Barium ions (Ba^2+^) can also produce strong alginate networks, although the resulting properties depend on the alginate composition, ion concentration, and gelation conditions. In contrast, magnesium ions (Mg^2+^) generally exhibit weaker effective crosslinking with alginate under comparable aqueous conditions and should not be considered equivalent to Ca^2+^ or Ba^2+^ as conventional alginate gelation agents. Consequently, Mg^2+^ alone may produce less robust networks or insufficient gelation under conditions that support Ca^2+^- or Ba^2+^-mediated gel formation (Table 1). However, the performance of magnesium-containing formulations must be interpreted according to their complete composition, particularly when modified alginate, additional polymers, or other crosslinking mechanisms are involved (Table 2) [47,48].

##### Polyelectrolyte Complexation or Electrostatic Crosslinking

**Figure 4:**
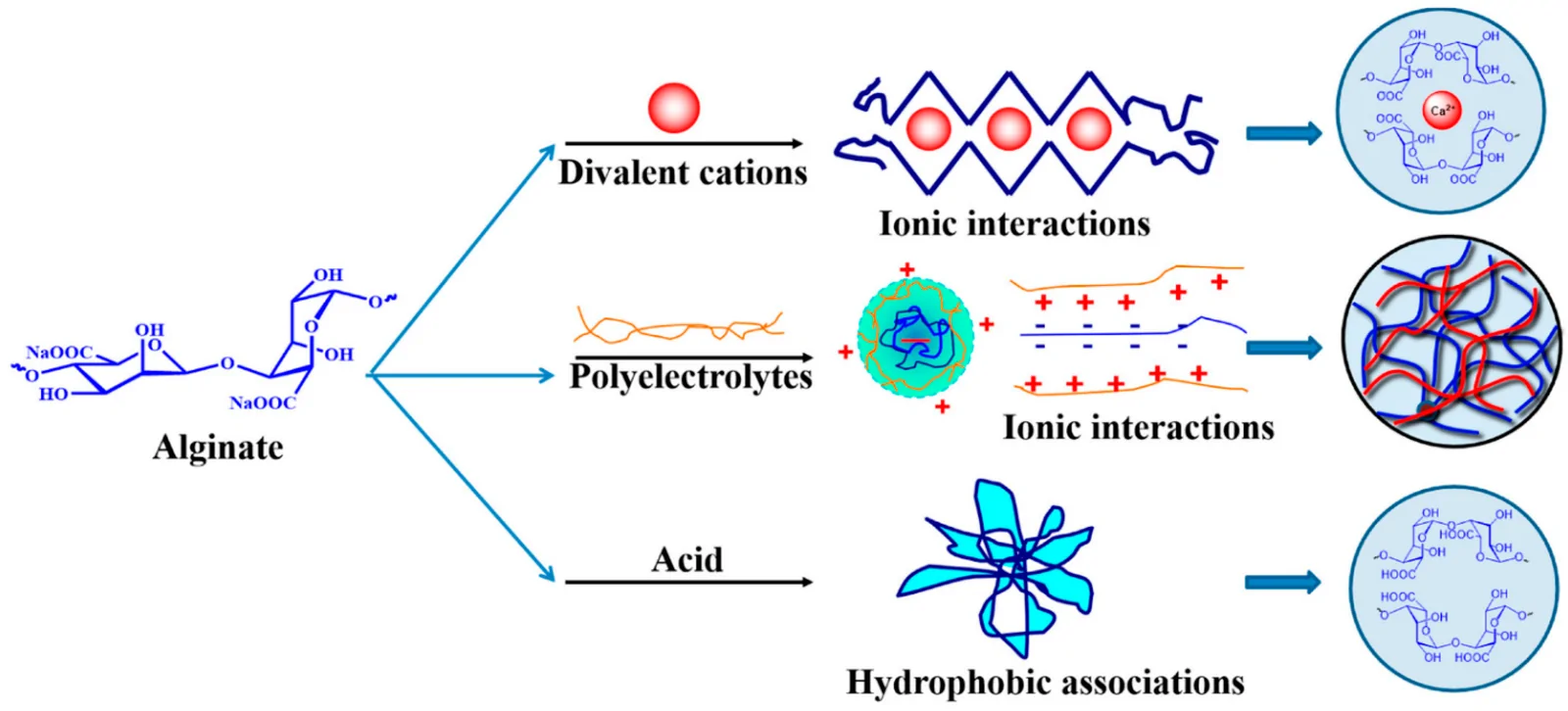
Physically crosslinked alginate hydrogel [44].

Another classic approach to form stable and reversible 3D networks is the electrostatic interaction between two oppositely charged polymers without requiring any external crosslinker, like a divalent cation (Figure 4). The stability of the formed electrolyte links depends on the pH of the system. A hydrogel scaffold is formed when the carboxylate group of alginate (anionic polymer) electrostatically interacts with the amino group of chitosan (cationic polymer) [49].

##### Hydrogen Bonding

**Figure 5:**
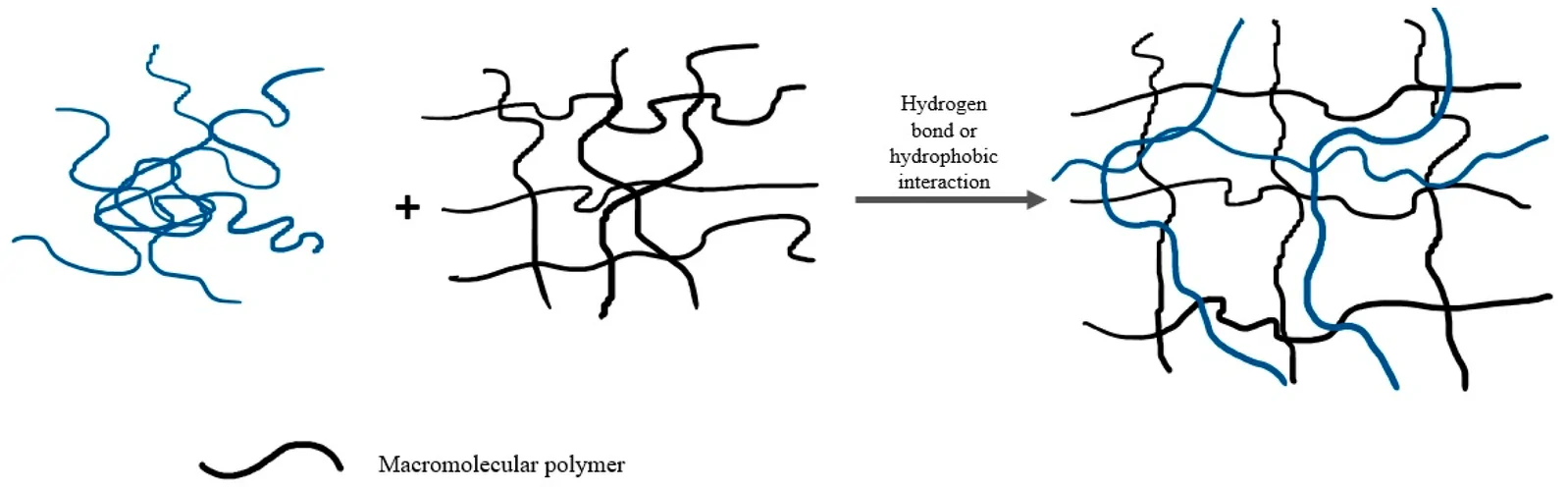
Hydrogen bonding crosslinking to form the alginate hydrogel [50].

Alginic acid hydrogel is formed due to hydrophobic association when the pH of the alginate solution is suddenly reduced. A sol–gel transformation occurs at a pH lower than the pKa value of the monomer. This hydrogel formation depends on the intermolecular hydrophobic association (Figure 4) and hydrogen bonding (Figure 5) [44,50].

**Table 1:** Summary of recent representative ionic crosslinking of alginate hydrogels [51].

Crosslinking Ion	Alginate Formulation	G-Block Interaction/Crosslinking Behaviour	Mechanical Property	Recovery (%)	Functional Outcome
Ca^2+^	Alginate formulation and concentration to be verified	Ca^2+^-mediated ionic gelation; interaction depends on alginate composition and crosslinking conditions	G′: range ofapproximately 0.02–0.03 Pa, as reported in the current table; unit and original data require verification	>86%; protocol and source require verification	Pseudoplastic and shear-thinning behaviour reported; confirm against the original study
Mg^2+^	ADA-GEL-1.0 Mg; not to be treated as unmodified alginate without verification	Reported enhancement associated with the modified formulation; do not interpret as evidence of strong gelation by Mg^2+^ alone	Compressive modulus: 33.9 ± 3.7 kPa, as reported in the current table; verify the original study		Proposed application in cartilage tissue engineering; supporting biological evidence requires verification
Sr^2+^	Alginate formulation and concentration to be verified	Ionic crosslinking reported; relative binding and network properties depend on experimental conditions	G′: range of approximately 0.04–0.05 Pa, as reported in the current table; unit and original data require verification	>86%; protocol and source require verification	Lower tan δ than the comparator is reported; verify the material and testing conditions
Ba^2+^	Alginate formulation and concentration to be verified	Ionic crosslinking reported; the resulting network depends on ion concentration and alginate composition	G′: range of approximately 0.08–0.10 Pa, as reported in the current table; unit and original data require verification	>86%; protocol and source require verification	Predominantly elastic response and filament consistency reported; verify against the original study
Cu^2+^	Alginate composite: composition and Cu^2+^ concentration to be specified	Ionic interactions reported in Cu–alginate systems; composite components must be identified	0.4–2.5 MPa range, as reported in the current table; the measured property and test conditions require verification		Antimicrobial activity and mechanical reinforcement reported for Cu–alginate composites; verify formulation-specific evidence
Zn^2+^	Alginate composite: composition and Zn^2+^ concentration to be specified	Ionic interactions reported in Zn–alginate systems; composite components must be identified	0.2–1.5 MPa range, as reported in the current table; the measured property and test conditions require verification		Antimicrobial activity reported for Zn–alginate composites; verify formulation-specific evidence
Fe^3+^	Alginate formulation and concentration to be verified	Rapid crosslinking reported; network uniformity and gelation depend on mixing and processing conditions	G′: range of approximately 0.02–0.03 Pa, as reported in the current table; unit and original data require verification	Approximately 35.3%; protocol and source require verification	Non-uniform network formation and limited recovery reported; verify against the original study
La^3+^	Alginate formulation and concentration to be verified	Rapid crosslinking reported; quantitative binding evidence is required	G′: range of approximately 0.10–0.15 Pa, as reported in the current table; unit and original data require verification	Approximately 33.4%; protocol and source require verification	Higher elastic response reported; verify against the original study
Cs^+^	Alginate formulation and concentration to be verified	No gel formation reported under the conditions described in the current table; conditions require verification	G″ ≫ G′, as reported in the current table; numerical values and test conditions are unavailable	Not applicable if no gel forms under the tested conditions	Liquid-like behaviour reported; verify against the original study

#### 4.1.2. Chemical Crosslinking Methods

In chemical crosslinking, a 3D network structure is generated due to covalent bonding between monomers or polymers. Due to the presence of a huge number of hydroxyl and carboxyl groups in the uronic acid units of the alginate chain, it can be further chemically reacted with other groups to form more stable, durable, and mechanically stronger alginate hydrogels [52,53].

##### Free Radical Polymerization

**Figure 6:**
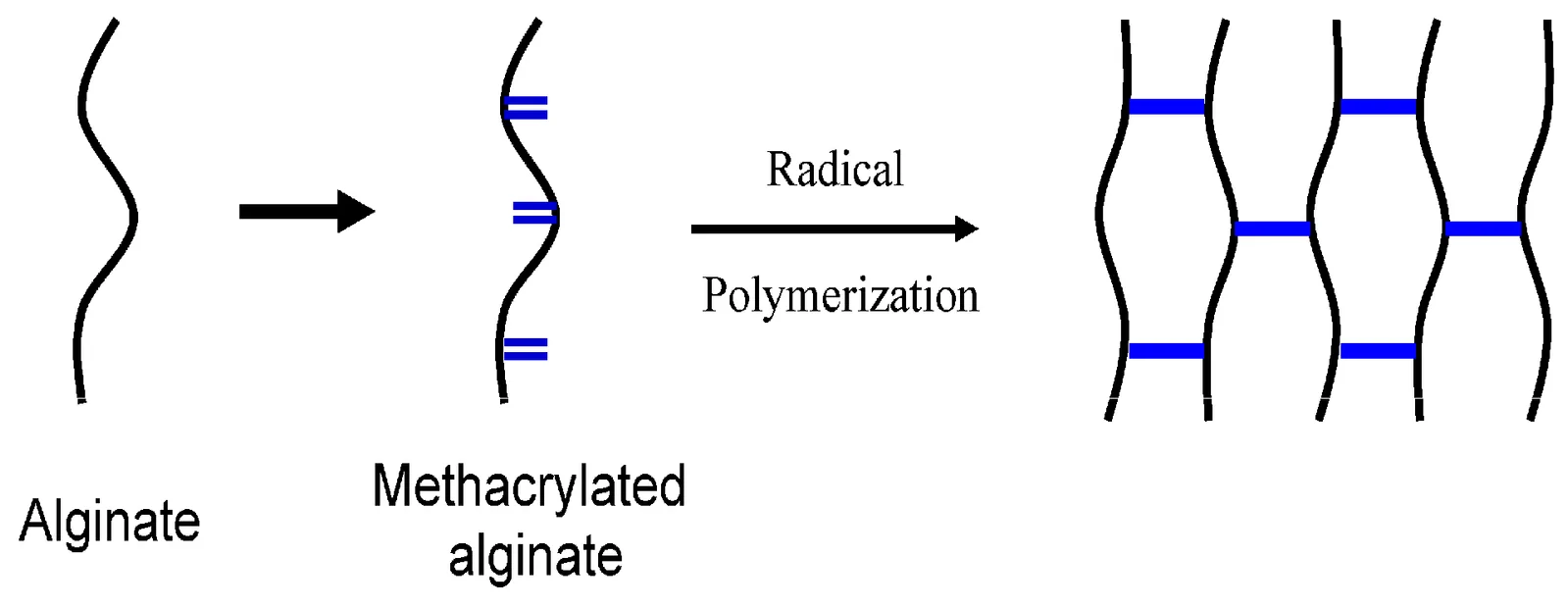
Schematic illustration of the preparation of methacrylated alginate and photocrosslinking of methacrylated alginate [41].

This method is used to convert linear polymers into 3D polymer networks where initiators are used to generate free radicals that induce the production of new free radicals on the linear polymer chain under specified conditions of pH and temperature [54]. According to Mohamed et al., the hydrogel made with natural methacrylate or acrylate polymers, such as methacrylate heparin, methacrylate gelatin, or methacrylate hyaluronic acid, has superior bioactivity. When stimuli are given, the resulting materials begin to crosslink [44,55]. Chemical initiators are employed in the free radical polymerisation process to transform a straight polymer into a three-dimensional polymer. This may be done at physiological temperatures and different pH levels. A mild gelation condition is required for the encapsulation of cells within the polymerized hydrogel and allows it to remain viable. Mixing of the derivatized selected macromer with the hydrogel and subsequent copolymerization are the unique features of this radical polymerization method. It was found that hydrogels made by free radical polymerisation of unsaturated C=C double-bond groups in methacrylate alginate are utilised as carriers of cells for tissue regeneration. Cell suspensions in a methacrylated alginate solution can be injected into the body and polymerised to create crosslinked alginate, which functions similarly to a tissue scaffold (Figure 6) [41].

##### Schiff-Base Reaction or Macromolecular Self-Crosslinking

**Figure 7:**
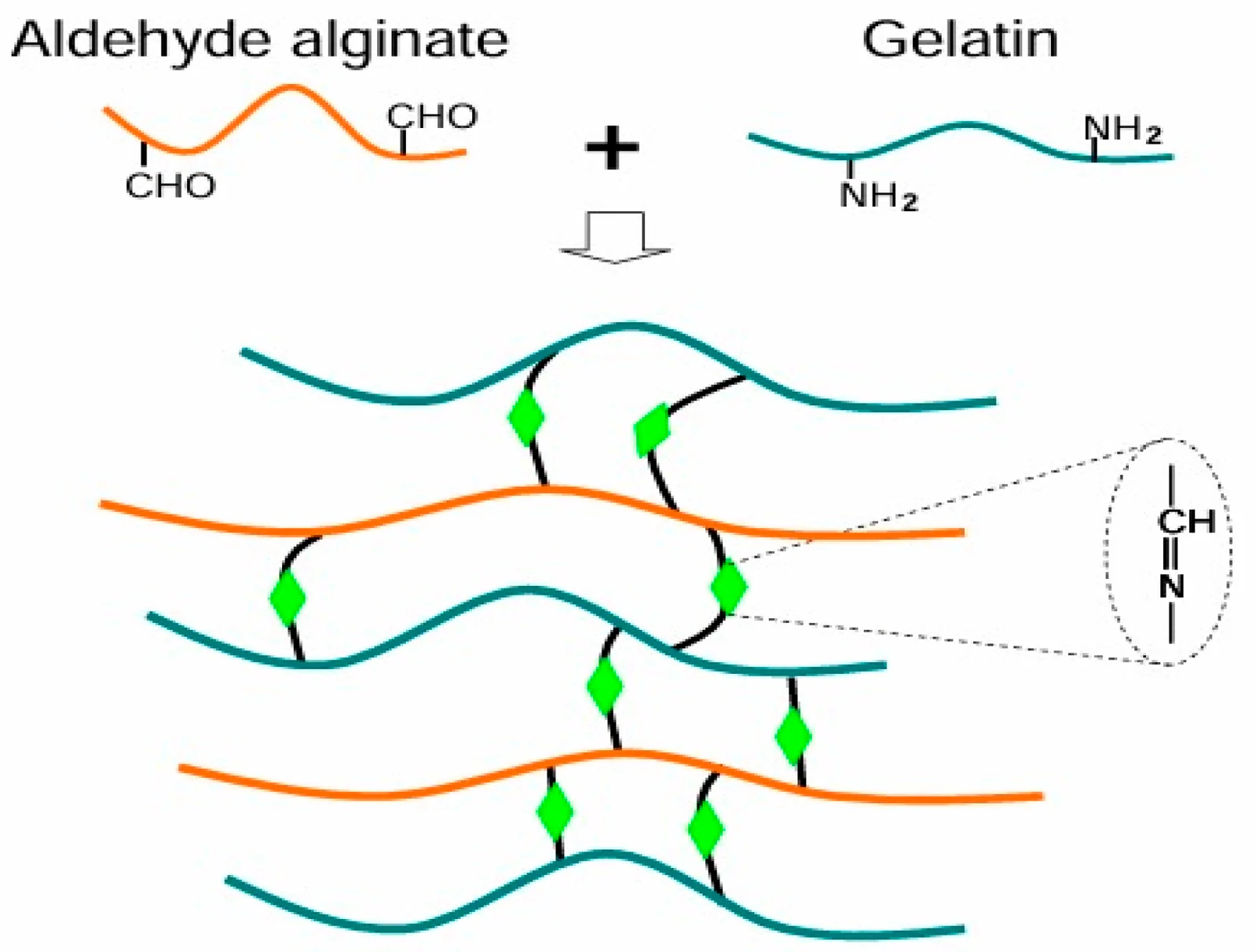
Scheme of alginate–gelatin composite hydrogel via Schiff-base reaction [41].

Schiff-base chemistry enables the formation of reversible pseudo-covalent imine linkages alongside permanent covalent bonds, resulting in hydrogels with dynamic and self-healing capabilities. Aldehyde groups of oxidised alginate react with amino groups of gelatin in a Schiff-base reaction that cleaves the carbon–carbon bonds of the cis-diol groups in the alginate’s molecular chain to produce reactive aldehyde functions through periodate oxidation, which can form chemical crosslinking with amino functions through Schiff-base linkage. Schiff-base bonding occurs between oxygenated alginate and biopolymers that contain amino groups, such as chitosan and collagen. Similar to this, polymers containing ketones or aldehydes react with a polymer containing hydroxylamine to generate oxime linkages and hydrogel. Despite not needing a catalyst, the reaction proceeds quickly. The interaction between the polymer’s hydroxyl or amino groups and isothiocyanate, which is used to make hydrogels, is a further illustration (Figure 7) [41]. Huan et al. reported an injectable self-healing hydrogel composed of oxidized sodium alginate (OSA) and carboxymethyl chitosan (CMCS), supplemented with VEGF (vascular endothelial growth factor) and HUVECs (human umbilical vein endothelial cells), which exhibited effective and prolonged glycemic control for 100 days in streptozotocin-induced diabetic mice [56].

**Table 2:** Crosslinking strategies for alginate hydrogels: mechanisms, performance determinants, limitations, and biomedical applications.

Parameter	Ionic Crosslinking	Physical Crosslinking	Chemical Crosslinking	Hybrid Crosslinking	References
Crosslinking mechanism	Ionic coordination between alginate carboxylate groups and multivalent cations, commonly Ca^2+^.	Physical interactions, including hydrogen bonding, electrostatic interactions, and polymer chain entanglement.	Covalent bond formation through reactions involving functionalized alginate and suitable crosslinkers.	Combination of ionic, physical, and/or covalent crosslinking mechanisms.	[51,53]
Representative formulation	Sodium alginate with an ionic crosslinker; identify the ion and formulation.	Alginate-based physically associated network; specify the actual formulation.	Oxidized or otherwise functionalized alginate with a compatible covalent crosslinker.	Alginate combined with a second polymer or crosslinking mechanism.	[51,53]
Material classification	Identify pure alginate or an alginate-based composite.	Identify pure alginate or an alginate-based composite.	Identify modified alginate or an alginate-based composite.	Identify all polymeric and non-polymeric components.	[51,53]
Crosslinking conditions	Report alginate concentration, ion concentration, pH, temperature, and gelation time.	Report polymer concentration, pH, temperature, and physical gelation conditions.	Report functionalization degree, crosslinker concentration, pH, temperature, and reaction time.	Report component concentrations and conditions for each crosslinking mechanism.	[51,53]
Swelling behavior	Report measured value with medium, pH, temperature, duration, and calculation method.	Report measured value and test conditions.	Report measured value and test conditions.	Report measured value and test conditions.	[51,53]
Mechanical characterization	Report tensile strength, compressive strength, and/or modulus as measured, with test conditions.	Report measured mechanical properties and test conditions.	Report measured mechanical properties and test conditions.	Report measured mechanical properties and test conditions.	[51,53]
Fatigue and structural stability	Report loading protocol and cycles to a defined endpoint, if evaluated.	Report loading protocol and cycles to a defined endpoint, if evaluated.	Report loading protocol and cycles to a defined endpoint, if evaluated.	Report loading protocol and cycles to a defined endpoint, if evaluated.	[51,53]
Degradation behavior	Report medium, temperature, duration, and mass-retention or degradation endpoint.	Report study-specific conditions and endpoint.	Report study-specific conditions and endpoint.	Report study-specific conditions and endpoint.	[51,53]
Biological evaluation	Report cell type, viability assay, time point, and control.	Report cell type, viability assay, time point, and control.	Report cell type, viability assay, time point, and control.	Report cell type, viability assay, time point, and control.	[51,53]
Advantages	Mild gelation conditions and suitability for cell encapsulation; stability depends on ion type and formulation.	May avoid some covalent crosslinking reagents; properties depend on physical interactions and formulation.	Can provide covalent network stabilization; residual reagents and reaction conditions require assessment.	Can combine mechanical stability, processability, and biological functionality.	[51,53]
Limitations	Ion exchange, swelling, and loss of structural integrity may occur under some conditions.	Physical interactions may be environment-sensitive and may provide insufficient stability in some formulations.	Potential cytotoxicity from residual reagents or initiators; additional chemical processing may be required.	More complex formulation, optimization, and characterization.	[51,53]
Representative applications	Cell encapsulation, drug delivery, and tissue-engineering scaffolds; cite specific studies.	Injectable or self-assembled systems and selected scaffold formulations; cite specific studies.	Scaffolds requiring covalent network stabilization; cite specific studies.	Multifunctional scaffolds requiring combined material properties; cite specific studies.	[51,53]

### 4.2. Nanofiber Scaffolds

Alginate nanofiber scaffolds are three-dimensional, highly porous fibrous materials derived from alginate, a naturally occurring anionic polysaccharide mainly obtained from brown seaweeds. Among the various techniques available for nanofiber fabrication, including self-assembly, freeze-drying, and temperature-induced phase separation, electrospinning stands out as a versatile approach for producing fibrous structures with tunable compositions, shapes, and morphologies [57,58].

#### 4.2.1. Electrospinning

**Figure 8:**
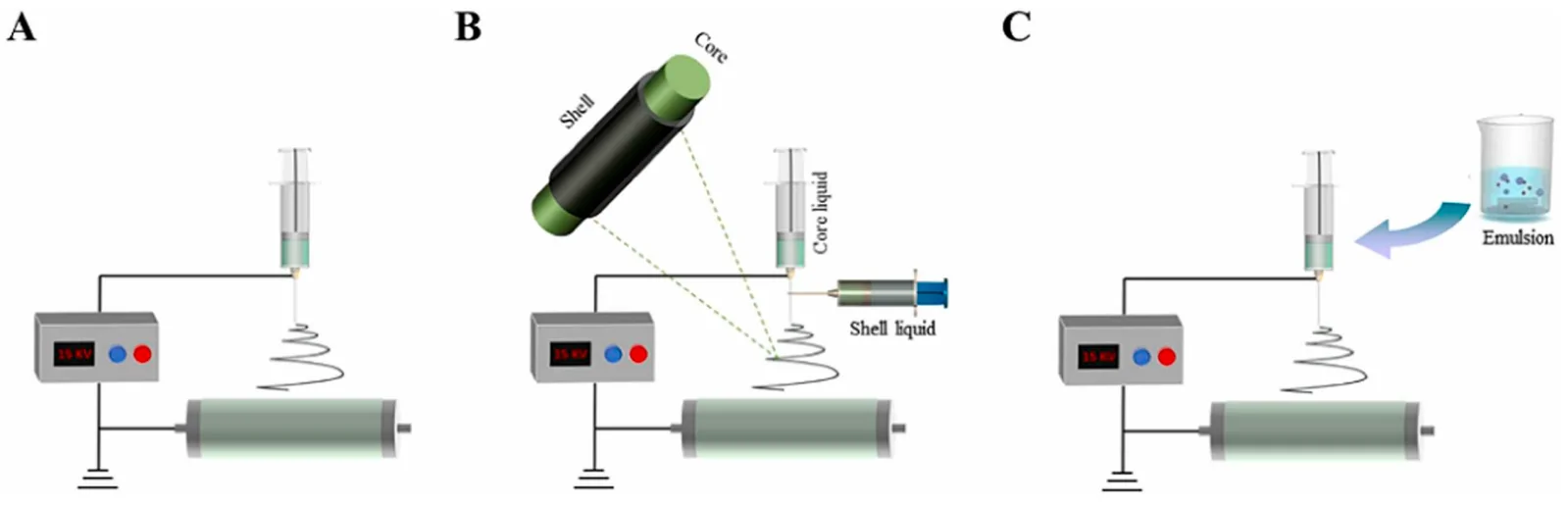
Electrospinning for fabrication of alginate nanofiber scaffold. (**A**) Blended electrospinning. (**B**) Coaxial electrospinning. (**C**) Emulsion electrospinning [64].

Modern electrospinning technology emerged in the 1990s as an efficient and cost-effective manufacturing technique that primarily transforms polymeric liquid systems into continuous nanoscale fiber structures [59]. Electrospun nanofibers are promising biomaterials with significant potential for a wide range of biomedical applications, including tissue engineering, wound healing, osteoporosis treatment, and the management of various other diseases. Alginate (AG) electrospinning has attracted considerable interest in biomedical applications due to its ability to produce fibers with a higher surface area than those obtained through conventional wet-spinning techniques. Nevertheless, the limited molecular entanglement and inherently rigid structure of alginate pose challenges in achieving continuous, uniform, and defect-free fibrous structures when electrospinning pure alginate solutions [60,61]. To facilitate the fabrication of alginate-based nanofibers, researchers have incorporated spinning-assisting or carrier polymers, such as poly(vinyl alcohol) (PVA) and poly(ethylene oxide) (PEO). However, several challenges remain, including the low alginate content, the presence of impurities within the resulting electrospun mats, and difficulties associated with the large-scale production of alginate-based nanofibers [62,63]. Researchers have overcome the poor spinnability of the material while simultaneously enhancing its tensile strength and antibacterial performance through the incorporation of other polymers using techniques such as blend electrospinning, coaxial electrospinning, and emulsion electrospinning (Figure 8) [64]. The ability to directly and continuously create nanofibers with a high specific surface area, high porosity, adjustable size, and simple surface customisation are some benefits of this technique. In addition, it is more scalable. Zheng et al. used wet spinning to combine sodium alginate with 2-methylimidazole zinc salt to create alginate composites fibres with superior mechanical qualities, high breaking strength, and antibacterial activity [65].

##### Blended Electrospinning

Blended electrospinning involves incorporating drugs or various solvents into a polymer solution and uniformly mixing them before the electrospinning process. The characteristics of the polymers and additives can influence the fiber diameter, morphology, distribution, and drug-release behavior. Polyethylene oxide (PEO), a synthetic linear polymer containing ether groups, can form strong hydrogen bonds with the hydroxyl groups of sodium alginate (SA), thereby enhancing the electrospinnability of SA [66,67]. Bassham et al. systematically investigated the effects of PEO content and surfactant interactions on the diameter and morphology of sodium alginate fibers. Their findings demonstrated that decreasing the PEO content while increasing the SA concentration caused the fiber morphology to transition from beads to continuous fibers and eventually to droplets, with the fiber diameter initially increasing before reaching a relatively stable value [68].

##### Coaxial Electrospinning

Coaxial electrospinning, also referred to as dual-fluid electrospinning, is a versatile technique for fabricating nanofibers with well-defined core–shell or hollow architectures by independently supplying two polymer solutions through a multi-needle system under a stable electric field. Since its introduction in 2002, the technique has undergone considerable development and has been widely employed in various biomedical and drug-delivery applications. In addition to enabling the simultaneous incorporation of multiple drugs into nanofibers, the distinctive core–shell structure provides effective separation of the inner drug from the external materials, thereby minimizing potential interactions and offering advantages for specialized applications [69,70,71,72]. Nista et al. successfully fabricated SA–CHI–PEO polyelectrolyte composite nanofibrous membranes using coaxial electrospinning, employing SA/PEO and chitosan (CHI)/PEO as the core and shell solutions, respectively. The resulting core–shell membranes exhibited good biocompatibility, mucoadhesive properties, and structural stability in aqueous environments, indicating their potential as controlled-release systems for localized oral drug delivery. However, further optimization of the electrospinning parameters was required to obtain a more uniform coaxial structure and consequently improve drug encapsulation efficiency and controlled-release performance [73].

##### Emulsion Electrospinning

Emulsion electrospinning technology is an innovative approach for fabricating core–shell nanofibers through uniaxial electrospinning of a stable emulsion system. Based on the characteristics of the dispersed and continuous phases, emulsion systems can generally be classified into two types: water-in-oil (W/O) and oil-in-water (O/W) emulsions. Under a high-voltage electrostatic field, the emulsifier-stabilized biphasic system undergoes pronounced phase-separation kinetics during Taylor cone formation. This phenomenon is primarily attributed to the differential solvent evaporation between the surface and core regions of the jet, which causes a rapid increase in the viscosity of the surface phase. Simultaneously, dispersed-phase droplets migrate towards the center of the jet under the combined influence of interfacial tension and electrorheological effects. During jet evolution, electric-field-induced stretching and the Lorentz force synergistically promote the axial orientation and elliptical deformation of the droplets. As solvent evaporation proceeds, followed by polymer solidification, nanofibers with well-defined core–shell interfaces are ultimately deposited onto the collector. A major advantage of this technology is its versatile drug-loading capability, enabling the simultaneous encapsulation of hydrophilic and hydrophobic functional substances while effectively preserving their molecular structures and biological activities. The resulting nanofibers demonstrate favorable sustained-release behavior and considerable potential for practical applications, owing to their relatively simple and convenient fabrication process [74,75,76]. Negahdari et al. fabricated an O/W emulsion electrospun core–shell mat using polycaprolactone (PCL) as the core material and a blend of sodium alginate (SA) and polyethylene oxide (PEO) as the shell-forming solution. The resulting nanofibrous mat exhibited excellent cytocompatibility and favorable mechanical characteristics, demonstrating considerable potential for various biomedical applications [77].

#### 4.2.2. Three-Dimensional Printing Technology

Tissue engineering and regenerative medicines are two areas where this approach is quite useful. Syringe extrusion 3D printing is the most popular 3D printing process and is frequently utilised in pharmaceutical applications. Alginate, gelatin, and other substances are examples of bioinks, which are materials utilised in syringe extrusion 3D printing. Three-dimensional printing technology allows for the fabrication of porous structures in tissue engineering. To pick bioinks in this technique, relevant raw materials are first chosen (Table 3) [78]. Alginate–cellulose nanofiber (ACNF) formulations have been created by R. Olmos-Juste et al. as appropriate bioinks for 3D printing technology. Curcumin was added to the bioinks, which were then 3D printed and freeze-dried to create porous nanofibrous scaffolds after their printability and shape fidelity were evaluated [79]. The benefit of 3D printing technology is that it can swiftly and reliably create tissue-engineered skin and generate very precise scaffolds with homogenous cell distribution. However, the size of the manufactured fibres is usually huge, and the variety of biological inks is not very diverse [78]. Beyond printability and shape fidelity, the performance of three-dimensionally printed alginate scaffolds should be evaluated through biological and functional endpoints, including cell viability, proliferation, phenotype maintenance, matrix deposition, tissue-specific differentiation, and integration with host tissue. Where bioactive agents are incorporated, release kinetics should be linked to the corresponding biological response. However, favorable printing characteristics alone do not establish regenerative efficacy. Comparative interpretation requires consideration of bioink composition, cell type, crosslinking strategy, culture conditions, and the duration and design of biological evaluation.

### 4.3. Microsphere Scaffolds

Alginate-based microsphere scaffold technology has significantly evolved in recent years, integrating advanced manufacturing methods and versatile bioengineering strategies for tissue regeneration and drug delivery. Sodium alginate is an anionic polysaccharide (brown-seaweed derived) that gels instantly in the presence of multivalent cations (most commonly Ca^2+^). This fast, mild ionic crosslinking, plus excellent cytocompatibility and tunable porosity, makes alginate a leading material for microsphere-based scaffolds used in cell encapsulation, growth-factor delivery, and modular (building-block) tissue constructs. However, native alginate has limited cell-adhesive motifs and modest mechanical strength, which has driven a wide array of fabrication and modification strategies [47].

#### 4.3.1. Extrusion/Ionic Gelation

**Figure 9:**
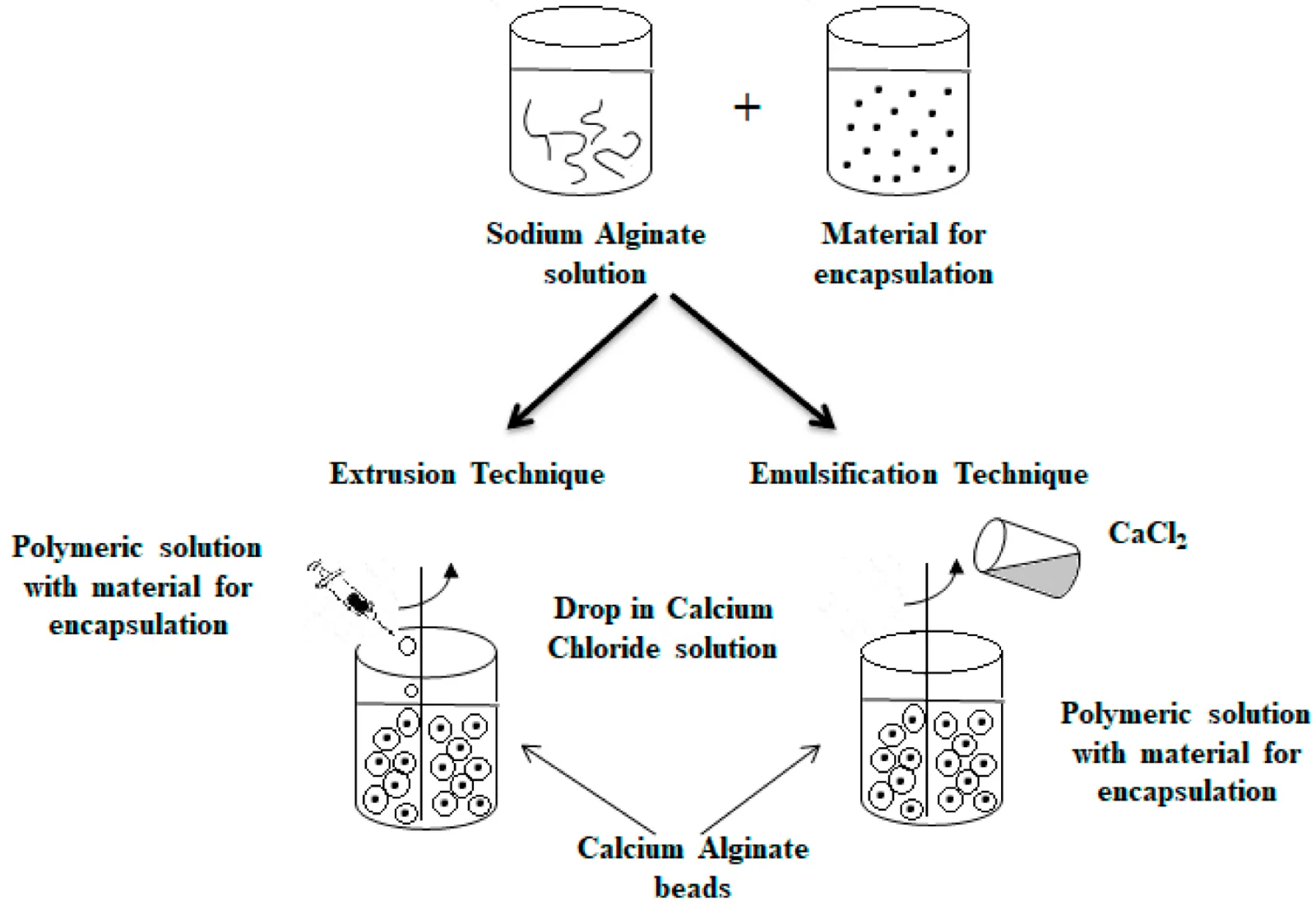
Schematic representation of extrusion (**left**) and emulsification (**right**) techniques [82].

This foundational method relies on crosslinking alginate (usually sodium alginate) with divalent cations like calcium (Ca^2+^) to yield stable gel microspheres [80,81]. The gelling process can be tuned for particle size and porosity. The technique is simple, cost effective, and easy scale-up for large beads. However, broad size distribution (unless using specialized nozzles) and lower control over monodispersity and internal microstructure compared with microfluidics are some limitations of this technique. It is commonly used in cell encapsulation and macro-porous scaffold formation (Figure 9) [47,82].

#### 4.3.2. Emulsification Technique

**Figure 10:**
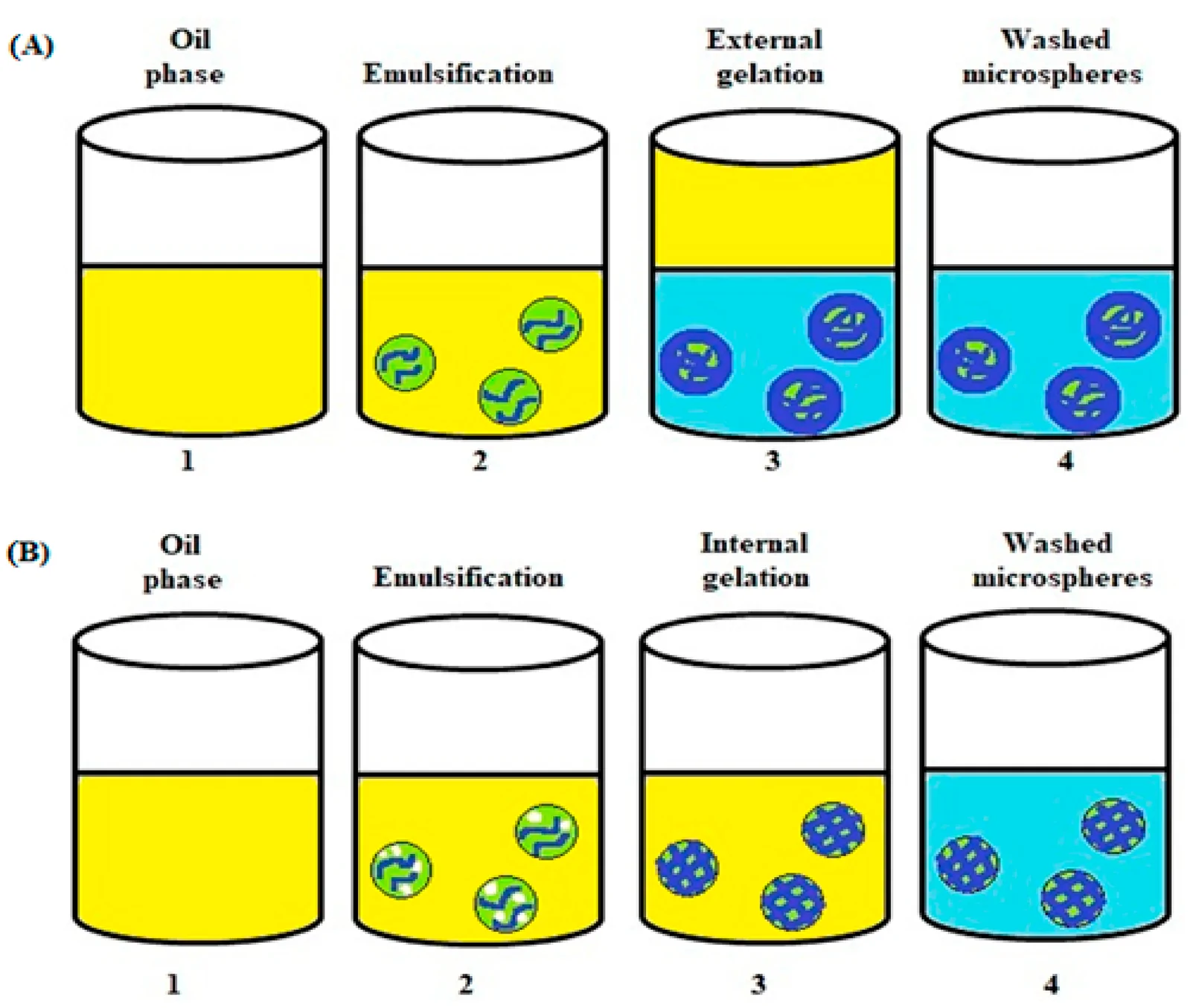
Alginate microsphere formation by the emulsification method—(**A**) external gelation and (**B**) internal gelation [82].

In modified emulsification, sodium alginate is dispersed in an oil phase, with surfactants and stirring to control size, followed by a cationic trigger for gelation (by adding Ca^2+^, either added to the aqueous phase before emulsification, or through Ca^2+^ diffusion, Figure 10) [83]. Key process variables such as alginate concentration, surfactant, and stirring speed precisely influence size and uniformity. The emulsification process is based on the mechanism of internal gelation and external gelation for alginate microspheres [84].

##### Internal Gelation

Internal gelation involves the incorporation of an insoluble calcium salt, typically calcium carbonate, into an aqueous polysaccharide phase. This phase is subsequently dispersed as droplets within a surfactant-stabilized oil phase. Upon acidification, with the pH decreasing from approximately 7.5 to 6.5, the calcium salt undergoes dissolution and releases Ca^2+^ ions. An oil-soluble organic acid, such as acetic acid, is commonly introduced into the continuous oil phase and diffuses rapidly across the oil–water interface. The rapid acidification of the aqueous droplets promotes Ca^2+^ release and consequently induces rapid crosslinking of alginate chains to form calcium alginate gel [85,86,87].

##### External Gelation

In external gelation, an alginate solution containing the encapsulated active substance is emulsified in an oil phase to form a W/O emulsion. A calcium chloride solution is subsequently added as the crosslinking agent. Ca^2+^ ions diffuse towards the surface of the dispersed alginate droplets, penetrate the aqueous phase, and crosslink the alginate chains, resulting in microsphere formation. The microspheres are then filtered and rinsed to eliminate residual oil. The resulting structures typically consist of a soft core enclosed within a rigid outer matrix [88,89].

#### 4.3.3. Spray-Drying Technique

**Figure 11:**
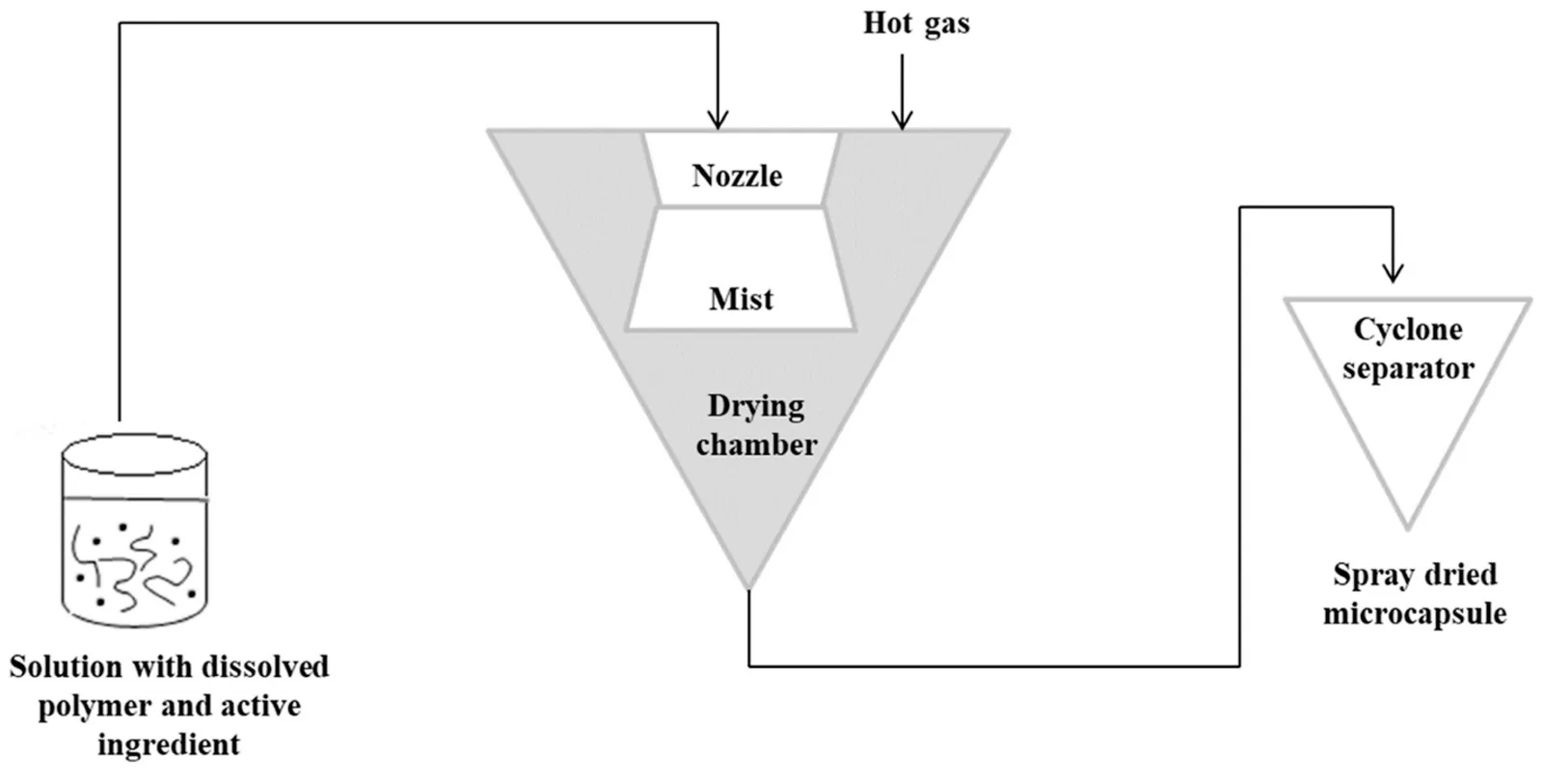
Spray-drying technique to prepare alginate microspheres [82].

The spray-drying technique is among the earliest methods employed for the preparation of alginate capsules (Figure 11). The fundamental concept of this technique was first documented in a patent by Samuel Percy in 1872 [90]. Although the process was commercially introduced during the 1920s, its large-scale application was not established until the early 1980s [91]. In spray drying, the active ingredient is initially incorporated into a polymer solution to form a homogeneous feed. This solution is subsequently pressurized and atomized into fine droplets, producing a mist within the drying chamber. Simultaneously, hot air or nitrogen is introduced into the chamber, facilitating the rapid evaporation of the solvent and formation of the capsules. The dried particles are subsequently conveyed to a cyclone separator for collection and recovery. Effective control of the feed rate, gas flow rate, and drying temperature is essential for achieving the desired product characteristics [92,93].

## 5. Biofunctionalization and Cell Interactions

Alginate scaffolds provide adjustable three-dimensional microenvironments for tissue engineering; nonetheless, they necessitate peptide functionalization, extracellular matrix mimicking, and ultra-pure modified chemistries to address weak adhesion and immunogenicity, while accommodating various cell types and enabling controlled bioactive cargo delivery. Recent studies indicate that RGD conjugation enhances islet and MSC functionality, ECM additives augment insulin secretion and angiogenic signals, and thorough depyrogenation reduces TLR-mediated inflammation; together, these advancements are advancing alginate-based systems towards clinically applicable, cell-instructive scaffolds with enhanced safety profiles [43].

### 5.1. Peptide Functionalization

Zhu et al. noted that alginate is devoid of natural cell-adhesive motifs; the conjugation of short ECM peptides reinstates integrin interaction and cellular viability without the limitations associated with full-length protein instability [94]. Medina et al. noted that RGD-functionalized alginate enhanced the viability of encapsulated islets and glucose-stimulated insulin release compared to unmodified alginate or scrambled RDG, whereas LRE, YIGSR, PDGEA, and PDSGR demonstrated inferior efficacy in that islet model, underscoring the necessity for cell- and ligand-specific design considerations [95]. Jha’s study indicates that Laminin-mimetic peptides such as YIGSR and IKVAV enhance neuronal and endothelial connections in engineered matrices; the selection of peptides, valency, and presentation density must align with target cell receptor profiles to optimize engraftment and functionality [96].

### 5.2. ECM Mimicry and Mechanics

Enck et al. demonstrated that the integration of a decellularized pancreatic extracellular matrix into alginate capsules improved human islet functionality, with stiffness and crosslinker selection further optimizing glucose responsiveness, highlighting the interplay of matrix mechanics and biochemical factors in microencapsulation design [97]. Nicolas and several researchers have discovered that basic ECM mimics prioritize scaffold chemistry, design, and ligand presentation to replicate adhesion, sequestration, and mechanotransduction observed in natural niches across several tissues [98]. In bone settings, Farjaminejad et al. noted that gelatin–alginate hybrids utilize gelatin’s RGD motifs alongside alginate’s printability to enhance osteoconductivity and chondrocyte gene expression when employed as bioinks or composite hydrogels [99].

### 5.3. Bioactive Cargos

Certain researchers demonstrated that alginate hydrogels and microspheres facilitate the prolonged release of proteins (e.g., BMP-2, TGF-β1) for osteochondral repair, enhancing mineralization and osteogenic differentiation both in vivo and in vitro. Furthermore, antioxidant nanoparticle (e.g., fullerenol) added to alginate improved stem cell viability in reactive oxygen species-rich myocardial infarction models through ERK/p38 activation and JNK inhibition, demonstrating a combinatorial therapeutic approach inside alginate matrices [43]. Ghasemi et al. highlight that oxygen carriers (e.g., PFOB) and angiogenic signals incorporated in alginate enhance oxygenation and vascularization in macro/microencapsulation formats for metabolically intensive grafts [100].

### 5.4. Cells: MSCs, Chondrocytes, Endothelial, Neural Progenitors, and Islets

Zhu et al. demonstrate that MSCs in RGD–alginate exhibit enhanced survival, increased production of pro-angiogenic factors, and superior ossification compared to adhesion-deficient alginate, hence endorsing their application in bone and vascular regeneration [94]. Farshidfar and colleagues noted that chondrocytes and mesenchymal stem cells benefit from double crosslinked alginate modified with cysteine and norbornene, which attain cartilage-like mechanical properties and enhance the production of SOX9, COL2A1, and ACAN in three-dimensional cultures and bioprinted constructions. Endothelial cells consistently attach to and proliferate on alginate composites (e.g., alginate dialdehyde–gelatin and collagen-enriched tubular gels), facilitating vascular graft sealing and endothelialization with regulated breakdown and hemocompatibility. Neural progenitors implanted in anisotropic, capillary-structured alginate scaffolds facilitate directional axon regeneration and integrate with spinal parenchyma without significant inflammation, indicating potential for directed neural repair [43].

### 5.5. Immune Interactions, Degradation, and Host Response to Alginate-Based Scaffolds

#### 5.5.1. Material Purity and Immunogenicity

The biological performance of alginate-based scaffolds is influenced not only by their chemical composition and crosslinking mechanism but also by the purity and quality of the starting material. Alginate extracted from different seaweed sources may contain residual proteins, polyphenols, endotoxins, and other impurities that can influence inflammatory responses following implantation. Inadequate purification and batch-to-batch variability may therefore contribute to inconsistent biological outcomes. Accordingly, the source, purification method, molecular weight, mannuronic-to-guluronic acid (M/G) ratio, and endotoxin content of alginate should be characterized wherever possible. These parameters are important for interpreting host responses and improving the reproducibility of alginate-based biomaterials [101,102].

#### 5.5.2. Foreign-Body Response and Immune Interactions

Following implantation, alginate-based scaffolds may interact with innate immune cells, including macrophages, which participate in material recognition, inflammatory signaling, tissue remodelling, and foreign-body responses. Depending on the material properties and implantation environment, persistent inflammatory reactions may contribute to foreign-body giant-cell formation and fibrous encapsulation. These responses can alter the local tissue microenvironment and compromise scaffold integration or the function of encapsulated cells. However, the magnitude and duration of the response cannot be attributed to alginate alone, because material purity, surface characteristics, scaffold architecture, crosslinking chemistry, and implantation site may all contribute. Evaluation of host compatibility should therefore consider inflammatory-cell recruitment, relevant cytokine or inflammatory markers, histological evidence of fibrosis, and tissue integration when these outcomes are available from the original studies [101].

#### 5.5.3. Crosslinking-Dependent Stability and Degradation

The crosslinking mechanism is a major determinant of alginate hydrogel stability, swelling behavior, and changes in mechanical integrity over time. Ionically crosslinked networks may undergo ion exchange in physiological environments, potentially resulting in swelling, weakening of the network, and loss of structural integrity. Covalently crosslinked networks may provide greater resistance to dissociation under particular conditions, although their persistence and degradation behavior depend on the crosslink chemistry, degree of modification, and surrounding environment. Hybrid systems combine multiple stabilization mechanisms, but their degradation cannot be predicted solely from the presence of more than one crosslinking mechanism. Consequently, the relative stability of different formulations should be established experimentally rather than inferred from crosslinking type alone.

#### 5.5.4. Distinguishing Degradation Endpoints

Degradation is frequently described using different measurements that should not be treated as equivalent. Mass loss measures a change in specimen mass, whereas swelling reflects fluid uptake and changes in dimensions. Loss of mechanical integrity describes a reduction in structural or load-bearing performance, while polymer degradation involves changes in the polymer itself. These processes may occur concurrently or at different rates. Therefore, studies comparing alginate-based scaffolds should specify the test medium, temperature, incubation duration, crosslinking conditions, and measurement method. Where available, mass retention, swelling ratio, mechanical-property retention, and degradation-related chemical changes should be reported separately. A degradation half-life should only be stated when it has been measured or estimated using an appropriate, explicitly described method.

#### 5.5.5. Tissue-Specific Degradation and Host Responses

The implications of degradation and immune interactions differ across tissue-engineering applications. In bone and cartilage engineering, scaffold stability and degradation must be considered in relation to tissue formation, matrix deposition, and the evolving mechanical requirements of the regenerating tissue. In skin repair, swelling, moisture retention, degradation, and interactions with the wound environment may influence scaffold performance. In pancreatic islet encapsulation, capsule integrity and long-term stability are particularly important because excessive swelling, loss of integrity, or pericapsular fibrotic overgrowth may compromise the transport of oxygen, nutrients, and secreted insulin. These outcomes should be interpreted according to the specific formulation and experimental model, distinguishing in vitro observations from animal studies and clinical evidence.

#### 5.5.6. Design Implications for Regenerative Medicine

The design of alginate-based scaffolds requires coordinated consideration of material purity, polymer composition, crosslink density, surface properties, mechanical stability, and degradation behavior (Table 4). Increasing network stability may improve structural persistence but does not necessarily improve biological performance or guarantee an appropriate host response. Similarly, faster degradation is not inherently advantageous if scaffold integrity is lost before sufficient tissue formation occurs. A suitable formulation should therefore balance structural support, transport properties, degradation behavior, and biological compatibility according to the target tissue and intended application. Standardized characterization and transparent reporting of material properties, degradation endpoints, and immune-related outcomes are essential for reliable cross-study comparison and the development of reproducible alginate-based regenerative systems [101,102,103].

Degradation and host responses in alginate-based scaffolds are strongly dependent on material composition, crosslinking chemistry, processing conditions, and the biological environment. Ionic crosslinking may permit ion exchange, swelling, and progressive loss of network integrity, whereas covalent crosslinking can modify network persistence and degradation behavior. Nevertheless, greater structural stability does not necessarily indicate superior biological performance, and mass loss alone does not establish complete polymer degradation. Host responses are also influenced by alginate purity, endotoxin contamination, residual processing reagents, scaffold architecture, and the implantation site. In pancreatic islet encapsulation, for example, pericapsular fibrosis may compromise transport between the surrounding tissue and encapsulated cells. Consequently, degradation and immunological outcomes should be evaluated using formulation-specific measurements and appropriate biological models rather than generalized rankings of crosslinking systems.

## 6. Tissue-Specific Applications of Alginate Scaffolds

There are two kinds of alginate scaffolds: injectable scaffolds and pre-formed scaffolds. For load-bearing applications, injectable scaffolds like hydrogel require the robust mechanical qualities generated by double networks, topological hydrogels, and composite hydrogels [47].

### 6.1. Bone Tissue Engineering

Alginate-based scaffolds, especially those made of hydrogels, have the potential to release the active ingredients and provide an environment that is favourable for bone regrowth. These properties include a very good porosity; a degradation rate or release profile that can be controlled by altering or adjusting the polymer crosslinking method, as well as other bone regrowth characteristics like osteoconductivity, osteoinductivity, osteogenic capacity, and osteocompatibility; and finally having osteogenic differentiation at the implantation site to make the regrowth bone structurally stable and mechanically robust [44]. Scaffold porosity and pore architecture are important determinants of nutrient transport, cell migration, and tissue ingrowth in bone tissue engineering. In alginate-based microparticle- and microfiber-aggregated scaffolds, swelling-associated changes in pore dimensions may improve the physical space available for cellular infiltration. However, increased pore width should not be interpreted as direct evidence of vascularization. Demonstrating vascular formation requires appropriate biological or histological evidence, such as endothelial-cell organization, vessel density, perfusion, or the presence of perfused microvessels in vivo. Therefore, the contribution of alginate scaffold architecture to vascularization should be interpreted in relation to the specific experimental outcomes reported in each study [11,104,105].

**Figure 12:**
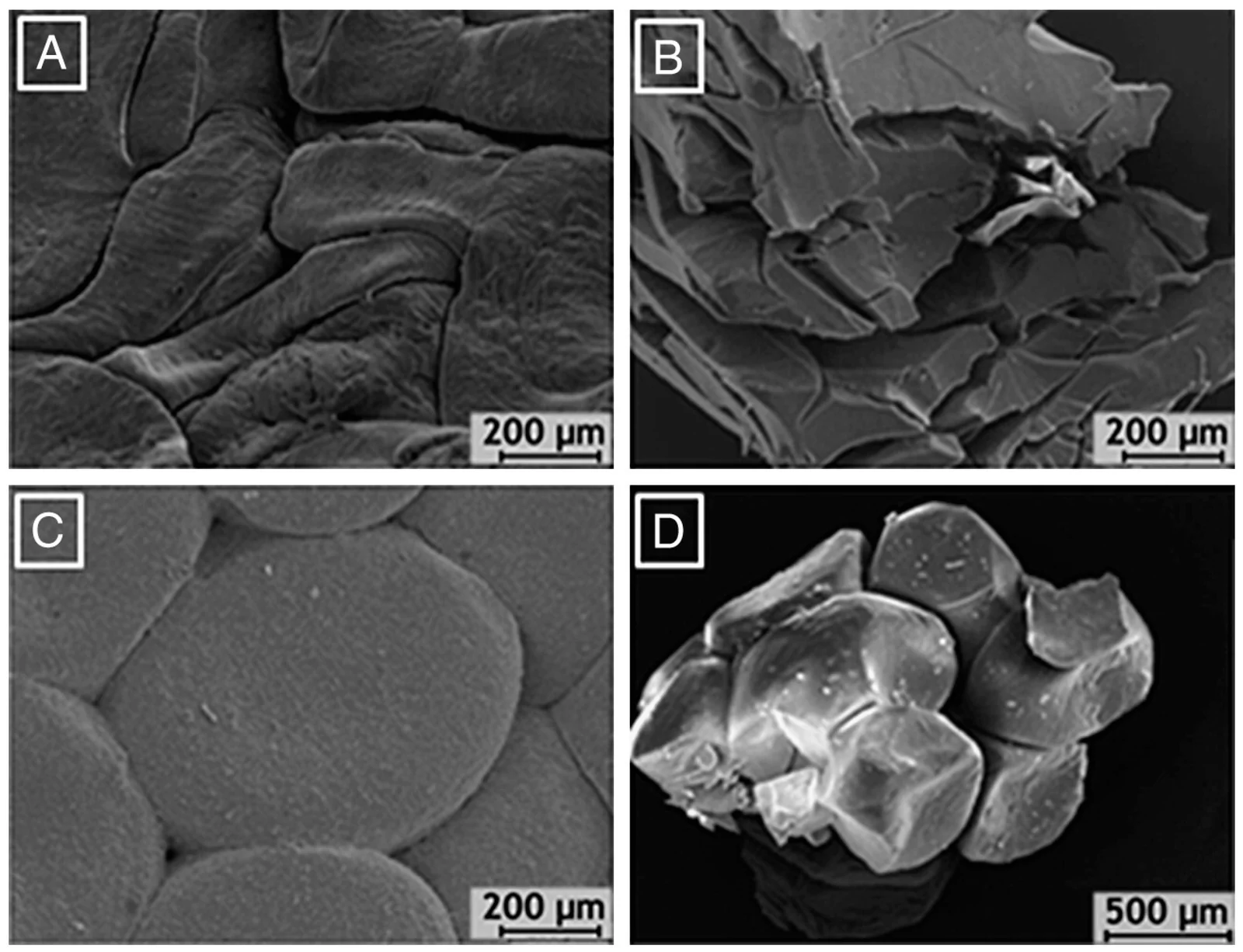
SEM images of the scaffolds produced: MF scaffold dried (**A**) and after being incubated in culture medium (**B**); MP scaffold dried (**C**) and after being incubated in culture medium (**D**). Reproduced from reference [104].

**Figure 13:**
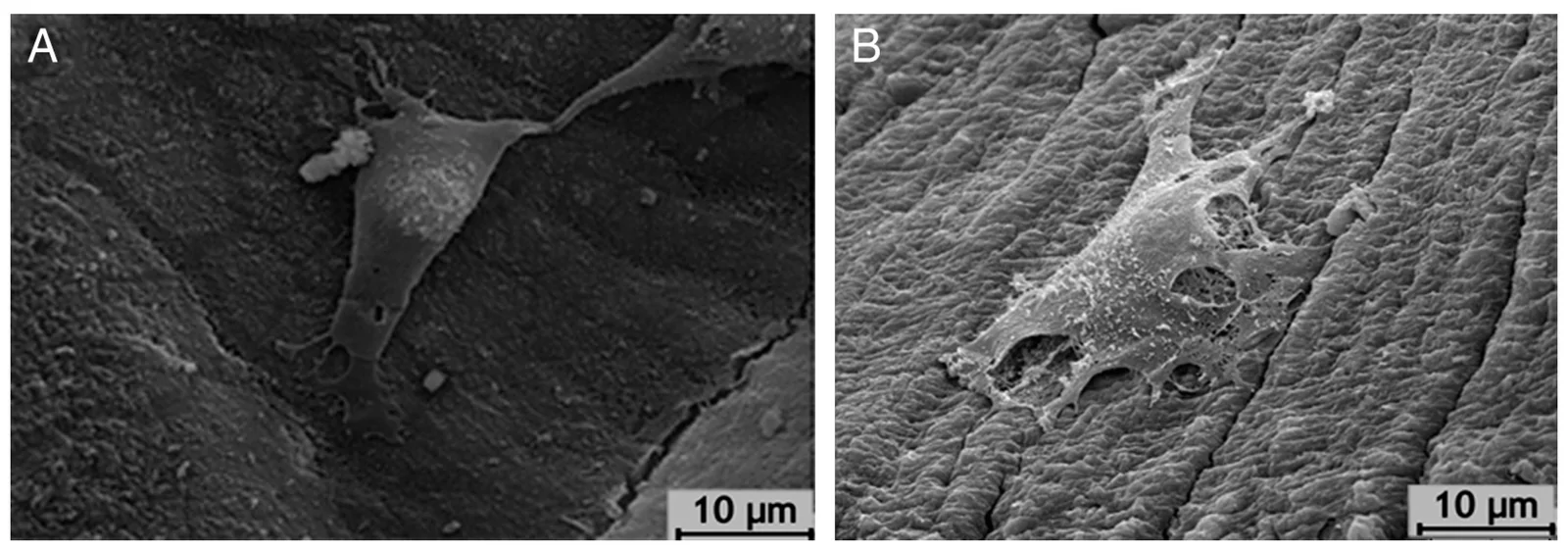
SEM images of cells adhered on the surface of the MP aggregated scaffold (2000×) (**A**) and on the surface of the MF aggregated scaffold (500×) (**B**). Reproduced from reference [104].

Alginate microparticles (MPs) and microfiber (MF)-aggregated scaffolds with excellent bone regeneration properties were created in this work in a simple, repeatable, and inexpensive manner (Figure 12 and Figure 13). Before the appliance of the scaffold in the BTE evaluation and characterisation of swelling properties, porosity, density, and mechanical properties, scanning electron microscopy (SEM) of the scaffold is very important for biocompatibility. SEM pictures demonstrated that when scaffolds were submerged in cell culture media, the pore width grew. Vascularization, oxygenation, cell migration, attachment, and proliferation biological processes essential to bone tissue regeneration will all be made possible by this porous framework. This swelling capacity is what causes the polymeric matrix to expand, increasing the size of the pores. Additionally, the scaffolds’ high swelling capacity caused a rise in pore width, indicating that they are appropriate for use in bone regeneration. In order to facilitate cell migration, tissue development, and even vascularization, an optimal scaffold for BTE application should have an average pore width of at least 100 μm, according to a morphological study. The mechanical study reveals that the scaffold begins to break down once it has begun to dehydrate, which is explained by the crosslinked polymer’s reduced packing of polymeric chains, which renders them more susceptible to oxidation which leads to breakdown. SEM analysis and an inverted light microscope were used to determine the scaffolds’ cytocompatibility. The MTS assay also verifies the cytotoxic profile if there is a notable difference between the positive control, the cell exposed to the scaffold, and the cell under the negative control, indicating that there was no major effect on the integrity or viability of the cells, which is crucial to their use in bone regeneration [104,105].

### 6.2. Cardiac Tissue Engineering

The leading cause of mortality globally is still acute myocardial infarction when blood flow to the coronary arteries is blocked, leading to necrosis, tissue remodelling, and fibrosis that can cause progressive cardiac damage and heart failure and the main problem is the low proliferation percentage for heart cell regeneration. The objective of cardiac tissue engineering is to develop a three-dimensional structure that can repair the injured heart by fusing cells with biocompatible materials. Adequate oxygen and nutrient transport is essential for maintaining the viability and function of encapsulated pancreatic islets. Alginate microcapsules can provide a protective environment for islet cells; however, the capsule itself does not establish vascular integration or neovascularization. Vascularization-related claims should therefore be supported by direct measurements, such as vessel density, perfusion, or histological evidence of vascular integration, where available. In the absence of such measurements, the discussion should focus on capsule stability, oxygen and nutrient transport, islet viability, and glucose-responsive insulin secretion rather than implying that vascularization has been demonstrated. Crosslinking and the manufacturing process were shown to improve the regeneration and contractility of cardiomyocytes. Gold, carbon, selenium, or silver nanoparticles can be added to scaffolds with low electrical conductivity to boost their conductivity to improve their biocompatibility. High swelling index and permeability are parameters that are closely linked to the degree of pore interconnectivity because they govern the flow of nutrients to the migrating cells inside the scaffold, resulting in effective cell development. It was shown that scaffolds with a sufficient pore size to promote cell infiltration and colonisation are extremely porous, a characteristic that directly affects cell penetration and nutrition and oxygen transport [106]. Graphene-based materials (GBMs) have been added to polymeric scaffolds that may be used as cardiac patches in a number of research studies to improve their mechanical and electrical conductivity. In the latest research, Baheirael et al. presented the creation of a number of freeze-dried alginate scaffolds that are covered with increasing concentrations of reduced graphene oxide (rGO) (between 5 and 30 mg/mL) for use as cardiac patches. SEM pictures demonstrated that the scaffolds’ pore sizes shrank as the rGO content increased, which was in line with the earlier research. Their conductive rGO-alginate (reduced graphene oxide alginate) scaffolds can be seeded or mixed with stem cells in the future to promote cardiomyocyte (CM) regeneration, reduce inflammation, and speed up angiogenesis [107,108].

### 6.3. Hepatic Cell Tissue Engineering

One of the body’s most essential and intricate organs, the liver coordinates detoxification, xenobiotic metabolism, glucose homeostasis, and immunological control [109]. In the United States alone, liver illnesses claim the lives of 26,000 persons annually at an estimated cost of USD 9 billion. Nowadays, scientists are concentrating on tissue engineering since liver transplantation is so expensive and there are few donor organs available [110]. Hepatocyte cells, the primary parenchymal cells in the liver, must be isolated and implanted into a biocompatible, biodegradable polymer system in order to create effective liver replacements. It has been extremely difficult to find a suitable method to adhere the cells to a scaffold matrix that can sustain the expression of genes particular to the liver [111]. HepG2 cells grow effectively on alginate scaffolds and express genes unique to the liver, according to Jeon et al. [112]. However, a major limitation of alginate is its lack of cell-adhesive motifs and its slow and poorly controlled degradation. To address these limitations, alginate can be chemically modified using metaperiodate to produce alginate dialdehyde (ADA). This modification introduces hydrolytically labile bonds into the alginate backbone, thereby improving its degradation behaviour [113].

Chen et al. proved that the combination of galactosylated chitosan and alginate dialdehyde/oxidized alginate promotes hepatocyte adhesion and proliferation [114]. Three-dimensional biomaterial-based tissue engineering offers a promising strategy for generating functional hepatic tissue to restore liver function in cases of liver failure. Mona et al. investigated the seeding and spatial distribution of cells within porous alginate scaffolds, a novel class of biomaterials designed for tissue-engineering applications. The hydrophilic properties, pore architecture, and interconnected structure of the alginate scaffolds facilitated effective hepatocyte incorporation, with approximately 70–90% of the initially introduced cells successfully seeded depending on the seeding technique employed. According to them, applying centrifugal force during seeding improves cell distribution in the porous scaffold, which in turn improves the seeding of cell suspensions. Within seven days, the number of viable hepatocytes declines to 33% of its initial value at a cell density of 0.28 × 10^−6^ cells/cm^−3^ scaffold; in denser cultures, the cells maintain higher viability while forming a network of connecting spheroids [5]. Hepatocyte degeneration occurred in vitro under the classical condition required to change the medium into HepatoZYME-SFM when the hepatocyte attached to the sodium alginate film to promote hepatocytes [7].

### 6.4. Pancreatic Islet Tissue Engineering

Alginate hydrogels, because of their rapid, mild ionic gelation, tunable mesh size, and relative biocompatibility, have been the dominant biomaterial platform for cell microencapsulation aiming to treat type 1 diabetes (T1D). Encapsulation seeks to provide immunoisolation (protecting implanted islets from host adaptive and innate immunity) while preserving rapid diffusive exchange of oxygen, glucose, insulin, and nutrients. In practice, reaching the simultaneous goals of immune protection, permselective transport, and long-term viability has proven challenging; success depends on alginate purity/composition, membrane architecture, oxygen management, and implantation strategy. Immunoisolation requires exclusion or mitigation of three principal threats: (1) cellular effectors (macrophages, T cells, and NK cells), (2) humoral effectors (complement and antibodies), and (3) foreign-body responses that lead to pericapsular fibrotic overgrowth (PFO).

Alginate capsules can physically exclude large immune cells and, when highly purified and properly surface-treated, reduce protein adsorption and local inflammatory activation. However, alginate networks are typically porous to soluble proteins and antibody fragments, so humoral components and small inflammatory mediators can still diffuse into the capsule microenvironment and damage encapsulated islets or prime external immune responses through antigen leakage [115,116,117]. Thus, alginate provides partial immunoisolation: strong protection against direct cellular cytotoxicity but incomplete protection from humoral or indirect inflammatory pathways unless combined with additional design measures.

Material strategies that improve immunoisolation include using ultra-pure, clinical-grade alginate to avoid innate immune activation; selecting intermediate G/M compositions that balance gel strength and reduced PFO; multilayer semipermeable membranes (e.g., alginate–poly-L-lysine–alginate, APA) to narrow effective pore size; and anti-fibrotic surface chemistries (PEGylation, zwitterionic, or sulfated alginates) to diminish protein adsorption and macrophage activation. These approaches have reduced macrophage recruitment and fibrosis in many preclinical studies but have not uniformly eliminated PFO in large animals or humans [116,118].

Permselectivity for encapsulated islets must achieve two contrasting requirements: extremely fast transport of small solutes (oxygen, glucose, and insulin) while limiting access of larger immune mediators and minimizing antigen escape. Because alginate hydrogels form hydrated meshes whose pore size depends on G/M content and crosslinking density, permselectivity is tunable but inherently limited: typical ionic alginate gels allow near-free diffusion of molecules up to several kDa, which is sufficient for insulin but not for completely blocking immunoglobulins or complement fragments [119,120]. Multilayer membranes and coatings (e.g., PLO/PLL layers and covalent grafts) can reduce effective pore sizes and selectively slow passage of macromolecules, but each additional layer increases mass-transfer resistance worsening oxygen and nutrient supply and increasing the risk of central hypoxia in larger capsules or macrocapsules [117,121].

Consequently, designers face a trade-off: microcapsules (~300–800 µm) maximize surface area to volume and thus minimize diffusion distances for oxygen and glucose, supporting islet viability, but they are prone to PFO and are not readily retrievable; macrocapsules or planar devices can be retrieved and engineered for vascularization/oxygenation, yet they impose larger diffusion distances and require specialized architectures (thin membranes and internal oxygenation) to avoid hypoxia [122,123]. Recent advances, such as perforated membranes, oxygen-carrying cores (perfluorocarbons), and continuous oxygenation systems, demonstrate improved function in large-animal models when combined with optimized permselective membranes, but these remain under active development.

To fine-tune this balance, multilayer microcapsules (e.g., alginate–poly-L-lysine–alginate) and covalently crosslinked or surface-modified alginate networks have been developed to control mesh size and surface charge. These approaches, combined with zwitterionic or sulfated modifications, minimize non-specific protein adsorption and attenuate pericapsular fibrotic overgrowth [116].

The regulatory landscape for alginate-based scaffolds is complex, varying depending on the scaffold’s intended use, whether as an acellular implant, a drug delivery matrix, or a cell-laden construct. In the United States, acellular alginate scaffolds are typically classified as medical devices (Class II or III), whereas cell-encapsulated systems fall under biologic-combination products requiring coordinated review by the FDA’s CBER (Center for Biologics Evaluation and Research) and CDRH (Center for Devices and Radiological Health). The European Medicines Agency (EMA) follows a similar hybrid categorization under Advanced Therapy Medicinal Products (ATMPs) [124]. Alginate-based biomaterials have progressed beyond laboratory research into selected clinical-development programmes, although the strength of evidence and regulatory status vary by product. Algisyl-LVR, an injectable calcium alginate hydrogel developed for left ventricular augmentation, was evaluated in the AUGMENT-HF randomized clinical trial involving 78 patients with advanced heart failure. The study assessed functional capacity and clinical outcomes, including peak oxygen consumption, over six months, with subsequent follow-up reported at one year [115]. DIABECELL, an alginate-encapsulated neonatal porcine islet-cell product investigated for type 1 diabetes, has also undergone human clinical evaluation. A published study reported outcomes in eight patients receiving encapsulated porcine islets, while a separate phase II study was registered to evaluate safety and preliminary efficacy [125]. These findings indicate clinical investigation rather than proof of established efficacy or regulatory approval. The current authorization and availability of each product should be confirmed from the relevant official regulatory authority and product sponsor before publication.

### 6.5. Cartilage Tissue Engineering

Alginate-based hydrogels have attracted considerable interest in cartilage tissue engineering because their hydrated three-dimensional networks can support chondrocyte encapsulation and cartilage matrix formation. Calcium-mediated ionic crosslinking enables hydrogel formation under relatively mild conditions, while alginate composition, polymer concentration, and crosslinking conditions influence scaffold stiffness, swelling, and stability. However, unmodified alginate lacks intrinsic cell-adhesive motifs and does not fully reproduce the biochemical and mechanical properties of native cartilage [126].

To improve scaffold performance, alginate has been investigated in combination with collagen, gelatin, hyaluronic acid, and other extracellular matrix-related components. Three-dimensional bioprinting can also help control scaffold architecture and cell distribution. Important evaluation parameters include cell viability, glycosaminoglycan and collagen production, cartilage-specific gene expression, mechanical properties, and integration with surrounding tissue [126,127,128].

Despite these advances, challenges remain in reproducing the structure and load-bearing properties of native cartilage, controlling scaffold degradation, and achieving stable integration with host tissue. Future research should emphasize standardized mechanical testing, long-term stability, and in vivo evaluation to establish the translational potential of alginate-based cartilage constructs [127,129].

### 6.6. Neural Tissue Engineering

Alginate-based scaffolds have been investigated for neural tissue engineering because their hydrated networks can provide a three-dimensional matrix for cell encapsulation and localized delivery of bioactive agents. Their mild gelation conditions and tunable physicochemical properties are relevant to neural cell culture and the development of biomaterial systems for neural repair. However, unmodified alginate lacks the intrinsic biochemical cues required for effective neuronal attachment, neurite extension, and functional neural network formation. To address these limitations, alginate can be combined with extracellular matrix components, adhesion-promoting peptides, or other bioactive materials. Laminin-mimetic peptides, such as IKVAV and YIGSR, may provide cell-recognition cues, while conductive composites may be useful for selected applications involving electrical signaling. Scaffold stiffness, architecture, degradation, and transport properties should be tailored to the intended neural application. Important evaluation parameters include neuronal viability, neurite extension, axonal organization, electrophysiological function, inflammatory response, and integration with host neural tissue. However, successful neural regeneration requires more than cell survival or neurite growth. Further research is needed to establish long-term functional recovery, appropriate host responses, and the safety of these scaffolds before clinical translation [130].

### 6.7. Skin Tissue Regeneration

**Figure 14:**
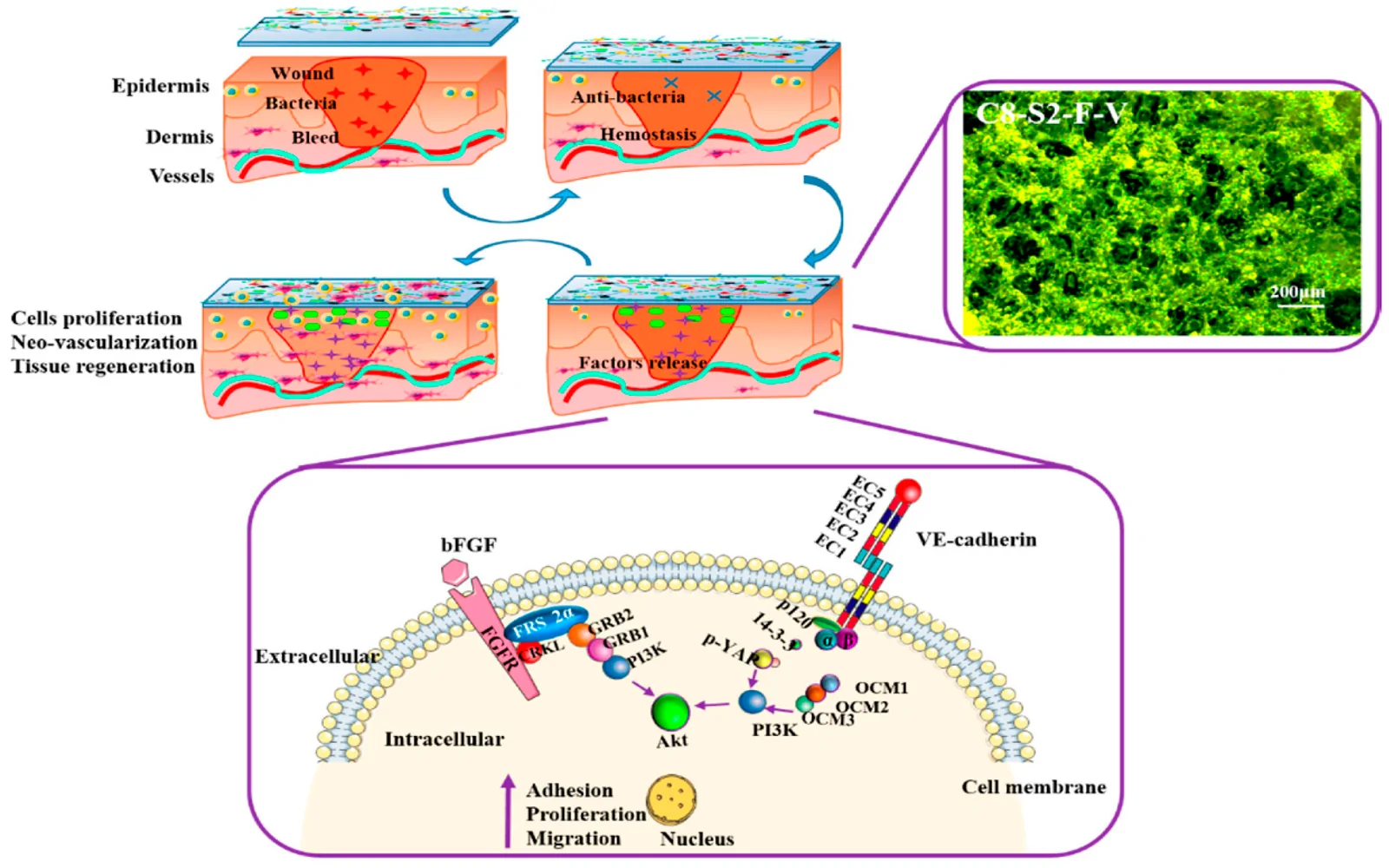
Relationship between hydrogels and wound healing process, involved in a cellular fluorescence image of the C8-S2-F-V hydrogel and the signalling pathways of FGF and VE-cadherin; CCM1, cerebral cavernous malformation protein 1; PI3K, phosphatidylinositol 3 kinases; YAP, yes-associated protein; FGFRs, FGF receptors [131].

**Figure 15:**
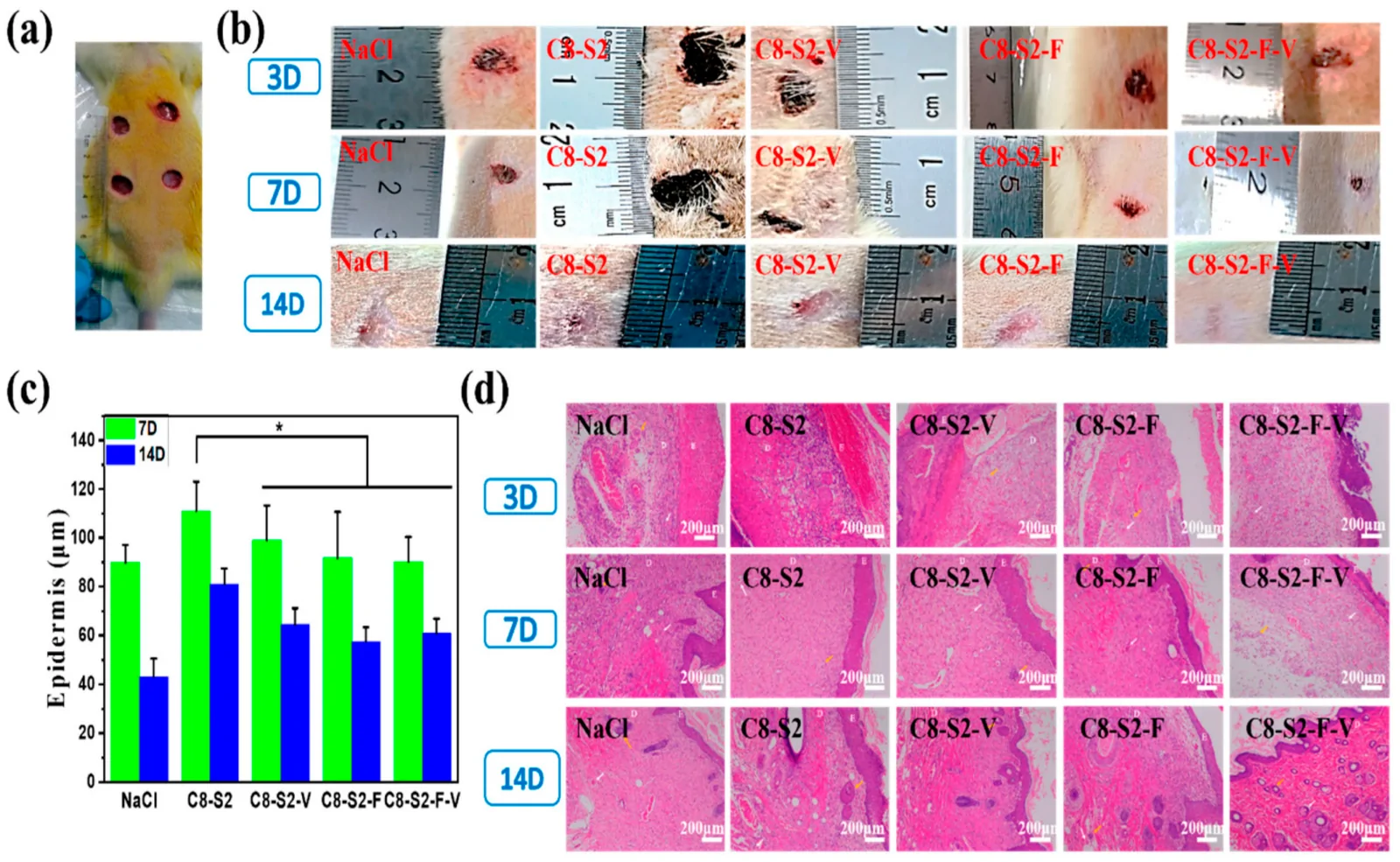
In vivo repair and regeneration evaluation of complex hydrogels. (**a**) Skin repair trauma experiments of complex hydrogels; (**b**) appearance of skin wound for 3, 7, and 14 days of observation for repair experiments; (**c**) epidermis thickness for different groups on 7th and 14th days (mean ± SD, *n* ≥ 3, * *p* < 0.05); (**d**) H&E staining of tissue sections of skin wound for 3, 7, and 14 days (blood vessels: white arrows; hair follicles: orange arrows; E: epidermal layer; and D: dermal layer) [131].

Alginate is widely used in skin tissue engineering because of its biocompatibility, moisture-retention capacity, and tunable gelation properties. However, unmodified alginate lacks intrinsic cell-adhesive motifs, limiting direct integrin-mediated attachment of fibroblasts and keratinocytes. Consequently, cell adhesion and spreading on alginate-based scaffolds depend on the material’s surface properties, biochemical functionalization, and incorporation of additional extracellular matrix components. Strategies such as RGD peptide conjugation and blending with gelatin, collagen, or other cell-adhesive biomaterials can provide adhesive cues that improve cellular attachment and spreading. Alginate-based hydrogels can also serve as carriers for cells and bioactive molecules, supporting localized delivery and the maintenance of a hydrated wound environment. Nevertheless, improvements in cell adhesion, proliferation, migration, and epithelialization should be attributed to the specific scaffold formulation and demonstrated experimental outcomes rather than to unmodified alginate alone (Figure 14 and Figure 15) [131,132,133,134,135].

Existing consensus recommendations have emphasized the importance of reporting alginate source, molecular weight, mannuronic-to-guluronic acid composition, material purity, and endotoxin content to improve reproducibility and comparability. Building on these recommendations, the present review organizes the reporting requirements within a processing–structure–function framework for alginate-based tissue-engineering scaffolds. This framework links material characterization and fabrication parameters to physicochemical properties, biological responses, and application-specific performance. In addition to established compositional descriptors, comparative evaluation should document crosslinker identity and concentration, polymer concentration, crosslinking conditions, scaffold architecture, mechanical testing protocols, degradation endpoints, and relevant biological outcomes. For cell-containing scaffolds, reporting should also specify cell type, viability assay, observation time, and appropriate controls. For vascularization and immune-response studies, the measured endpoints and experimental model should be clearly identified. These recommendations are intended to facilitate transparent study comparison and evidence interpretation rather than to replace existing consensus guidelines. Their practical contribution lies in integrating established reporting standards with application-specific characterization and biological evaluation across alginate scaffold systems [136,137,138,139].

## 7. Critical Gaps, Challenges, and Future Directions

Alginate is a versatile, clinically attractive hydrogel for tissue engineering, but its translation is limited by reproducibility, incomplete mechanistic standards, and fragmented comparability between studies.

A persistent barrier is simple: not all alginates are the same. Natural variability (seaweed species, harvest season, extraction, and purification methods) produces wide differences in molecular weight, block structure (G/M ratio), and impurity load (proteins, polyphenols, and endotoxin). These differences translate directly into divergent gelation kinetics, mechanical properties, and host responses (e.g., pericapsular fibrosis). Yet many manuscripts omit critical material descriptors (exact source, supplier/lot, measured M/G, and endotoxin levels), making cross-study comparison and meta-analysis difficult [131,132]. Controversy also exists around which compositional metrics to report. The community has emphasized reporting at least alginate supplier/lot, extraction/purification method, number-average molecular weight (Mn or Mw), G/M ratio (measured by NMR), and endotoxin units per mg. Adoption of these minimal reporting standards would reduce irreproducibility and would allow a mechanistic interpretation of results [133,134].

Currently, claims such as “stiff” or “soft” alginate are used loosely. Tissue engineering needs quantitative, standard benchmarks that map scaffold properties to biological outcomes. Suggested core metrics (and measurement methods) include (i) elastic modulus (compressive and shear; reported with strain rate and geometry) and stress-relaxation times (creep/relaxation testing); (ii) mesh size estimates or mesh-related parameters (from swelling and polymer concentration) to predict diffusivity for proteins and growth factors; (iii) degradation half-life (hydrolytic and ion-exchange conditions) and in vitro enzymatic susceptibility where relevant (e.g., alginate lyase); (iv) biochemical quality: G/M ratio, Mw, endotoxin (EU/mg), and residual protein/polyphenol content; and (v) biological readouts: standardized cell viability (live/dead at defined depths), metabolic function (e.g., insulin secretion for islets), and immune readouts (macrophage activation, PFO scoring in a defined implantation model). A harmonized benchmark matrix, analogous to what the mechano-biology field has proposed for synthetic hydrogels, would enable head-to-head comparisons and data pooling for meta-analysis [140].

## 8. Conclusions

Alginate-based scaffolds represent versatile biomaterials with clinical applications, engineered through various methods, including ionic, covalent, and dual crosslinking, as well as composite and peptide-functionalized designs. These scaffolds provide cell-instructive mechanics, tunable degradation, and optimized mass transport for various tissues. They also mitigate native limitations in adhesion, strength, and immunogenicity by utilizing RGD/ECM ligands, ultra-pure chemistries, and smart degradable linkers to facilitate predictable remodelling. Integrating micro/nanofabrication techniques such as electrospinning, microfluidics, and bioprinting with hierarchical porosity and controlled release of bioactive cargos (e.g., BMP-2, TGF-β, and oxygen carriers) enables alginate platforms to facilitate vascularization, stress-relaxation guided mechano-biology, and lineage-specific outcomes in applications related to bone, cartilage, cardiac, neural, hepatic, skin, and islet tissues. These advances may help move alginate-based scaffolds beyond proof-of-concept towards improved translational relevance and manufacturability. However, durable clinical impact will depend on reproducibility, controlled host responses, standardized material characterization (including molecular weight, mannuronic-to-guluronic acid ratio, and endotoxin content), validated sterilization protocols, harmonized mechanical and transport testing, and appropriate long-term in vivo studies. Although alginate-based scaffolds demonstrate promising potential for cartilage and neural tissue engineering, further preclinical validation and long-term functional studies are required to establish their safety, efficacy, and translational potential. Advances in immunomodulatory chemistry, organoid-compatible systems, and AI-guided design may further support the development of alginate-based platforms for future clinical applications.

## Figures and Tables

**Table 3:** Biological and functional evaluation of alginate-based bioinks and composite scaffolds for tissue engineering.

Scaffold Design	Biological or Functional Outcome	Important Limitation
Alginate–gelatin bioink	Cell viability, proliferation, differentiation, or tissue-specific markers.	Gelatin content and crosslinking affect performance.
Alginate–cellulose nanofiber scaffold	Cell compatibility, pore architecture, shape fidelity, and any reported functional outcome.	Do not equate printability with tissue regeneration.
Alginate–collagen composite	Cell adhesion, matrix deposition, and tissue-specific maturation.	Outcomes depend on composition and cell type.
Growth-factor-loaded alginate bioink	Release kinetics and relevant biological response.	Release alone does not establish therapeutic efficacy.

**Table 4:** Degradation and host-response characteristics of alginate-based scaffolds across tissue-engineering applications.

Scaffold or Crosslinking System	Degradation-Related Parameters	Tissue-Engineering Application	Host-Response Considerations
Ionically crosslinked alginate	Swelling, mass loss, changes in mechanical integrity, and stability over time	Bone, cartilage, and soft-tissue engineering	Ion exchange may weaken the network; response depends on ion type, material purity, and implantation conditions
Covalently crosslinked alginate	Mass loss, network stability, and changes in mechanical properties over time	Tissue-specific scaffolds requiring prolonged structural support	Crosslinker chemistry, residual reagents, and degradation products require assessment
Alginate–polymer composites	Changes in mass, swelling, mechanical integrity, and degradation of the complete formulation	Skin repair and other tissue-engineering applications	Response depends on the alginate, additional polymers, additives, and material purity
Alginate microcapsules	Capsule integrity, permeability, swelling, and long-term stability	Pancreatic islet encapsulation	Pericapsular fibrotic overgrowth may impair transport and cellular function
Modified or biofunctionalized alginate	Degradation and mechanical stability of the modified formulation	Tissue-specific regenerative scaffolds	Functionalization may alter protein interactions, cellular responses, and material persistence

## Data Availability

No new data were created or analyzed in this study. Data sharing is not applicable to this article.

## References

[1] Tomic S.L., Babic R.M.M., Vukovic J.S., Filipovic V.V., Nikodinovic-Runic J., Vukomanovic M. (2023). Alginate-based hydrogels and scaffolds for biomedical applications. Mar. Drugs.

[2] Akhtar Z. (2024). Exploring biomedical engineering (BME): Advances within accelerated computing and regenerative medicine for a computational and medical science perspective exploration analysis. J. Emerg. Med. Open Access.

[3] Grijalvo S., Nieto-Díaz M., Maza R.M., Eritja R., Díaz D.D. (2019). Alginate hydrogels as scaffolds and delivery systems to repair the damaged spinal cord. Biotechnol. J..

[4] Angelozzi M., Miotto M., Penolazzi L., Mazzitelli S., Keane T., Badylak S.F., Piva R., Nastruzzi C. (2015). Composite ECM–alginate microfibers produced by microfluidics as scaffolds with biomineralization potential. Mater. Sci. Eng. C.

[5] Mona D.G., Iris G.B., Riad A., Smadar C. (2003). Liver tissue engineering within alginate scaffolds: Effects of cell-seeding density on hepatocyte viability, morphology, and function. Tissue Eng..

[6] Mansouri N., Al-Sarawi S.F., Mazumdar J., Losic D. (2019). Advancing fabrication and properties of three-dimensional graphene–alginate scaffolds for application in neural tissue engineering. RSC Adv..

[7] Slack J.M.W. (2025). Stem Cell.

[8] Chan B.P., Leong K.W. (2008). Scaffolding in tissue engineering: General approaches and tissue-specific considerations. Eur. Spine J..

[9] Chng V.J.Y., Wan A.C.A. (2023). The scaffold concept for alternative proteins. J. Food Eng..

[10] Almeida H.V., Sathy B.N., Dudurych I., Buckley C.T., O’Brien F.J., Kelly D.J. (2017). Anisotropic shape-memory alginate scaffolds functionalized with either type I or type II collagen for cartilage tissue engineering. Tissue Eng. Part A.

[11] Sahoo D.R., Biswal T. (2021). Alginate and its application to tissue engineering. SN Appl. Sci..

[12] Sharma A., Gupta M.N. (2002). Three phase partitioning of carbohydrate polymers: Separation and purification of alginates. Carbohydr. Polym..

[13] Biswas R., Kapat P., Ghosh A. (2025). Pharmaceutical Polymers in Drug Delivery: An Overview. Int. J. Pharm. Pharm. Sci..

[14] Bouhadir K.H., Lee K.Y., Alsberg E., Damm K.L., Anderson K.W., Mooney D.J. (2001). Degradation of partially oxidized alginate and its potential application for tissue engineering. Biotechnol. Prog..

[15] Fernando I.S., Kim D., Nah J.W., Jeon Y.J. (2019). Advances in functionalizing fucoidans and alginates (bio) polymers by structural modifications: A review. Chem. Eng. J..

[16] Smidsrød O., Skjåk-Braek G. (1990). Alginate as immobilization matrix for cells. Trends Biotechnol..

[17] Rinaudo M. (2008). Main properties and current applications of some polysaccharides as biomaterials. Polym. Int..

[18] Wei Q., Zhou J., An Y., Li M., Zhang J., Yang S. (2023). Modification, 3D printing process and application of sodium alginate based hydrogels in soft tissue engineering: A review. Int. J. Biol. Macromol..

[19] Zhang M., Zhao X. (2020). Alginate hydrogel dressings for advanced wound management. Int. J. Biol. Macromol..

[20] Venkatesan J., Bhatnagar I., Manivasagan P., Kang K.H., Kim S.K. (2015). Alginate composites for bone tissue engineering: A review. Int. J. Biol. Macromol..

[21] Rhein-Knudsen N., Ale M.T., Ajalloueian F., Meyer A.S. (2017). Characterization of alginates from Ghanaian brown seaweeds: *Sargassum* spp. and *Padina* spp.. Food Hydrocoll..

[22] Andersen T., Strand B.L., Formo K., Alsberg E., Christensen B.E. (2011). Alginates as biomaterials in tissue engineering. Carbohydrate Chemistry.

[23] Grant G.T., Morris E.R., Rees D.A., Smith P.J.C., Thom D. (1973). Biological interactions between polysaccharides and divalent cations: The egg-box model. FEBS Lett..

[24] Kavand A., Noverraz F., Gerber-Lemaire S. (2024). Recent advances in alginate-based hydrogels for cell transplantation applications. Pharmaceutics.

[25] Datta S. (2023). Advantage of Alginate Bioinks in Biofabrication for Various Tissue Engineering Applications. Int. J. Polym. Sci..

[26] Cao Y., Cong H., Yu B., Shen Y. (2023). A review on the synthesis and development of alginate hydrogels for wound therapy. J. Mater. Chem. B.

[27] Rana M.M., Das S., Ghosh A., Mandal T. (2024). Evolution of Hybrid Hydrogels: Next-Generation Biomaterials for Drug Delivery and Tissue Engineering. Gels.

[28] Li R., Zhao Y., Zheng Z., Liu Y., Song S., Song L. (2023). Bioinks adapted for in situ bioprinting scenarios of defect sites: A review. RSC Adv..

[29] Castillo T., Flores C., Salgado-Lugo H., Peña C.F., Galindo E. (2023). Alginate and γ-polyglutamic acid hydrogels: Microbial production strategies and biomedical applications. A review of recent literature. Electron. J. Biotechnol..

[30] Zhang W., Kuss M., Yan Y., Shi W. (2023). Dynamic Alginate Hydrogel as an Antioxidative Bioink for Bioprinting. Gels.

[31] Naeimipour S., Boroojeni F.R., Lifwergren P., Selegård R., Aili D. (2023). Multimodal and dynamic cross-linking of modular thiolated alginate-based bioinks. Mater. Today Adv..

[32] Keshavarz M., Jahanshahi M., Hasany M., Kadumudi F.B., Mehrali M., Shahbazi M.A. (2023). Smart alginate inks for tissue engineering applications. Mater. Today Bio.

[33] Hu T., Fang J., Shen Y., Li M., Wang B., Xu Z. (2024). Advances of naturally derived biomedical polymers in tissue engineering. Front Chem..

[34] Al-Roujayee A.S., Hilaj E., Deepak A., Jyothi S.R., Hamid J.A., Ariffin I.A., Garg A. (2025). Alginate-based systems: Advancements in drug delivery and wound healing. Int. J. Polym. Mat. Polym. Biomater..

[35] Iqbal Y., Amin F., Usman Y., Farrukh Sarfraz M. (2024). Alginate-based hydrogels with inorganic nanomaterials: A promising approach for wound healing and bone tissue regeneration. Eur. Polym. J..

[36] Mathur V., Agarwal P., Kasturi M., Srinivasan V., Seetharam R.N., Vasanthan K.S. (2025). Innovative bioinks for 3D bioprinting: Exploring technological potential and regulatory challenges. J. Tissue Eng..

[37] Xu F., Dawson C., Lamb M., Mueller E., Stefanek E., Akbari M., Hoare T. (2022). Hydrogels for tissue engineering: Addressing key design needs towards clinical translation. Front. Bioeng. Biotechnol..

[38] Konwarh R., Singh A.P., Varadarajan V., Cho W.C. (2024). Harnessing alginate-based nanocomposites as nucleic acid/gene delivery platforms to address diverse biomedical issues: A progressive review. Carbohydr. Polym. Technol. Appl..

[39] Fitriani N. (2025). Alginate-based hydrogels with amniotic membrane stem cells for wound dressings application. Stem Cells Cloning Adv. Appl..

[40] Cota Quintero J.L., Ramos-Payán R., Romero-Quintana J.G., Ayala-Ham A., Bermúdez M., Aguilar-Medina E.M. (2025). Hydrogel-Based Scaffolds: Advancing Bone Regeneration Through Tissue Engineering. Gels.

[41] Sun J., Tan H. (2013). Alginate-Based Biomaterials for Regenerative Medicine Applications. Materials.

[42] Raus R.A., Wan Nawawi W.M.F.W., Nasaruddin R.R. (2021). Alginate and alginate composites for biomedical applications. Asian J. Pharm. Sci..

[43] Farshidfar N., Iravani S., Varma R.S. (2023). Alginate-Based Biomaterials in Tissue Engineering and Regenerative Medicine. Mar. Drugs.

[44] Chen X., Wu T., Bu Y., Yan H., Lin Q. (2024). Fabrication and Biomedical Application of Alginate Composite Hydrogels in Bone Tissue Engineering: A Review. Int. J. Mol. Sci..

[45] Lianqi C., Wei L., Analucia M., Katsuyoshi N., Yapeng F. (2020). Egg-box model-based gelation of alginate and pectin: A review. Carbohydr. Polym..

[46] Brax M., Schaumann G.E., Diehl D. (2019). Gel formation mechanism and gel properties controlled by Ca^2+^ in chia seed mucilage and model substances. J. Plant Nutr. Soil Sci..

[47] Zhang H., Cheng J., Ao Q. (2021). Preparation of Alginate-Based Biomaterials and Their Applications in Biomedicine. Mar. Drugs.

[48] Hernández-González A.C., Téllez-Jurado L., Rodríguez-Lorenzo L.M. (2020). Alginate hydrogels for bone tissue engineering, from injectables to bioprinting: A review. Carbohydr. Polym..

[49] El-Sherbiny I.M., Yacoub M.H. (2013). Hydrogel scaffolds for tissue engineering: Progress and challenges. Glob. Cardiol. Sci. Pract..

[50] Radulescu D.M., Neacsu I.A., Grumezescu A.M., Andronescu E. (2022). New Insights of Scaffolds Based on Hydrogels in Tissue Engineering. Polymers.

[51] Liang X., Chen S., Liang Y., Wang M., Wang Q., Chen D., Ma X., Ding H., Zhong H.-J. (2026). Alginate-Based Hydrogels: Recent Progress in Preparation, Property Tuning, and Multifunctional Applications. Gels.

[52] Gao Y., Peng K., Mitragotri S. (2021). Covalently Crosslinked Hydrogels via Step-Growth Reactions: Crosslinking Chemistries, Polymers, and Clinical Impact. Adv. Mater..

[53] Junyan T., Yuning L., Yuqiong G., Yue Z., Xinying L., Dingxilei L., Xinyi L., Yang L. (2023). Development of alginate-based hydrogels: Crosslinking strategies and biomedical applications. Int. J. Biol. Macro..

[54] Choi J.R., Yong K.W., Choi J.Y., Cowie A.C. (2019). Recent advances in photo-crosslinkable hydrogels for biomedical applications. Biotechniques.

[55] Jeon O., Bouhadir K.H., Mansour J.M., Alsberg E. (2009). Photocrosslinked alginate hydrogels with tunable biodegradation rates and mechanical properties. Biomaterials.

[56] Huan Z., Li J., Cao X., Lu M., Yu Y., Li L. (2025). Injectable self-healing hydrogels loaded with islet-integrated microfiber scaffolds for islet transplantation in diabetes mellitus. Sci. Bull..

[57] Azimi B., Maleki H., Zavagna L., de la Ossa J.G., Linari S., Lazzeri A., Danti S. (2020). Bio-based electrospun fibers for wound healing. J. Funct. Biomater..

[58] Riaz T., Khenoussi N., Rata D.M., Atanase L.I., Adolphe D.C., Delaite C. (2023). Blend electrospinning of poly(ε-caprolactone) and poly(ethylene glycol-400) nanofibers loaded with ibuprofen as a potential drug delivery system for wound dressings. AUTEX Res. J..

[59] He J., Chen Y., Wang J., Chen S., Lan X., Liu M., Chen X., Ou W., Wang H. (2025). Lavender Essential Oil-Loaded Composite Fiber Membrane Prepared by Coaxial Electrospinning for High-Performance, Antibacterial, and Lasting-Fragrance Air Filtration. J. Appl. Polym. Sci..

[60] Raţă D.M., Cadinoiu A.N., Grădinaru L.M., Fuioaga P.C., Vochita G., Delaite C., Atanase L.I. (2025). Design and characterization of curcumin-loaded electrospun nanofibers based on poly(vinyl alcohol) and sodium alginate. Express Polym. Lett..

[61] Zdiri K., Cayla A., Elamri A., Erard A., Salaun F. (2022). Alginate-based bio-composites and their potential appli cations. J. Funct. Biomater..

[62] Taemeh M.A., Shiravandi A., Korayem M.A., Daemi H. (2020). Fabrication challenges and trends in biomedical applications of alginate electrospun nanofibers. Carbohydr. Polym..

[63] Bhardwaj N., Kundu S.C. (2010). Electrospinning: A fascinating fiber fabrication technique. Biotechnol. Adv..

[64] Wang T., Su E. (2024). Electrospinning meets food packaging: A promising pathway towards novel opportunities in food preservation. Food Packag. Shelf.

[65] Zheng X., Zhang Y., Zou L., Wang Y., Zhou X., Yao L., Wang Z., Li C., Qiu Y. (2020). Robust ZIF-8/alginate fibers for the durable and highly effective antibacterial textiles. Colloids Surf. B Biointerfaces.

[66] Henning S., Lach R., Schönherr H., Longo R., Catauro M., Vertuccio L., Guadagno L. (2023). Comparison between Morphological and Mechanical Properties of Membranes Produced via Coaxial and Monoaxial Electrospinning. Macromol. Symp..

[67] Zhou X., Wang Y., Ji C. (2025). Advances in Preparation and Biomedical Applications of Sodium Alginate-Based Electrospun Nanofibers. Gels.

[68] Carroll-Bassham H., Brettmann B.K. (2024). The interplay between conductivity and viscosity in electrospinning charged biopolymers. MRS Commun..

[69] Wang H., Ren R., Li M., Gao Z., Li H., Li S. (2025). Coaxial emulsion electrospinning fiber films loaded with OEO for active packaging of pork. Food Res. Int..

[70] Chen X., Wu J., Zhang T., Li H., Zhang R., Lu W., Guo Y. (2025). Multifunctional coaxial gelatin/polylactic acid three-dimensional nanofibrous scaffolds for diabetic wounds. Int. J. Biol. Macromol..

[71] Ji E., Song Y.H., Lee J.K., Kim Y., Lee E., Joo K.I., Seo J.H. (2025). Bioadhesive levan-based coaxial nanofibrous membranes with enhanced cell adhesion and mesenchymal stem cell differentiation. Carbohyd. Polym..

[72] Sun Z., Zussman E., Yarin A.L., Wendorff J.H., Greiner A. (2003). Compound Core–Shell Polymer Nanofibers by Co-Electrospinning. Adv. Mater..

[73] Nista S.V.G., Bettini J., Mei L.H.I. (2015). Coaxial nanofibers of chitosan–alginate–PEO polycomplex obtained by electrospinning. Carbohyd. Polym..

[74] Walden R., Aazem I., Hinder S., Brennan B., Goswami A., McGranaghan G., Pillai S.C. (2024). Parametric optimisation of PDMS/PMMA nanofibers prepared using emulsion electrospinning technique. Results Mater..

[75] Lv H., Wang C., Xu E., Jin Z., Zhao H., Yuan C., Zhao M., Yu B., Wu Z., He D. (2025). Preparation of starch-based oral fast-disintegrating nanofiber mats for astaxanthin encapsulation and delivery via emulsion electrospinning. Int. J. Biol. Macromol..

[76] Zhang C., Li S., Chen D., Lu W., Xiao C. (2025). Emulsion-coaxial electrospinning: The role of zein as a shell layer in multicore-shell structured nanofibers for bioactive delivery. Int. J. Biol. Macromol..

[77] Negahdari N., Alizadeh S., Majidi J., Saeed M., Ghadimi T., Tahermanesh K., Arabsorkhi-Mishabi A., Pezeshki-Modaress M. (2024). Heat-treated alginate-polycaprolactone core-shell nanofibers by emulsion electrospinning process for biomedical applications. Int. J. Biol. Macromol..

[78] Jiang Z., Zheng Z., Yu S., Gao Y., Ma J., Huang L., Yang L. (2023). Nanofiber Scaffolds as Drug Delivery Systems Promoting Wound Healing. Pharmaceutics.

[79] Olmos-Juste R., Alonso-Lerma B., Pérez-Jiménez R., Gabilondo N., Eceiza A. (2021). 3D printed alginate-cellulose nanofibers based patches for local curcumin administration. Carbohydr. Polym..

[80] Asgari S., Pourjavadi A., Licht T.R., Boisen A., Ajalloueian F. (2020). Polymeric Carriers for Enhanced Delivery of Probiotics. Adv. Drug Deliv. Rev..

[81] Ching S.H., Bansal N., Bhandari B. (2017). Alginate gel particles–A review of production techniques and physical properties. Crit. Rev. Food Sci. Nutr..

[82] Łętocha A., Miastkowska M., Sikora E. (2022). Preparation and Characteristics of Alginate Microparticles for Food, Pharmaceutical and Cosmetic Applications. Polymers.

[83] Liao J., Wang B., Huang Y., Qu Y., Peng J., Qian Z. (2017). Injectable alginate hydrogel cross-linked by calcium gluconate-loaded porous microspheres for cartilage tissue engineering. ACS Omega.

[84] Chan L.W., Lee H.Y., Heng P.W.S. (2006). Mechanisms of External and Internal Gelation and Their Impact on the Functions of Alginate as a Coat and Delivery System. Carbohydr. Polym..

[85] Paques J.P. (2015). Alginate Nanospheres Prepared by Internal or External Gelation with Nanoparticles. Microencapsulation and Microspheres for Food Applications.

[86] Choukaife H., Doolaanea A.A., Alfatama M. (2020). Alginate Nanoformulation: Influence of Process and Selected Variables. Pharmaceuticals.

[87] Paques J.P., Van Der Linden E., Van Rijn C.J.M., Sagis L.M.C. (2014). Preparation Methods of Alginate Nanoparticles. Adv. Colloid Interface Sci..

[88] Mahdi M.H., Diryak R., Kontogiorgos V., Morris G.A., Smith A.M. (2016). In Situ Rheological Measurements of the External Gelation of Alginate. Food Hydrocoll..

[89] Kaklamani G., Cheneler D., Grover L.M., Adams M.J., Bowen J. (2014). Mechanical Properties of Alginate Hydrogels Manufactured Using External Gelation. J. Mech. Behav. Biomed. Mater..

[90] Percy S.R. (1872). Improvement in Drying and Concentrating Liquid Substances by Atomizing.

[91] Piñón-Balderrama C.I., Leyva-Porras C., Terán-Figueroa Y., Espinosa-Solís V., Álvarez-Salas C., Saavedra-Leos M.Z. (2020). Encapsulation of Active Ingredients in Food Industry by Spray-Drying and Nano Spray-Drying Technologies. Processes.

[92] Martín M.J., Lara-Villoslada F., Ruiz M.A., Morales M.E. (2015). Microencapsulation of Bacteria: A Review of Different Technologies and Their Impact on the Probiotic Effects. Innov. Food Sci. Emerg. Technol..

[93] Keshani S., Daud W.R.W., Nourouzi M.M., Namvar F., Ghasemi M. (2015). Spray Drying: An Overview on Wall Deposition, Process and Modeling. J. Food Eng..

[94] Zhu F., Nie G., Liu C. (2023). Engineered biomaterials in stem cell-based regenerative medicine. Life Med..

[95] Medina J.D., Alexander M., Hunckler M.D., Fernández-Yagüe M.A., Coronel M.M., Smink A.M., Lakey J.R., De Vos P., García A.J. (2020). Functionalization of Alginate with Extracellular Matrix Peptides Enhances Viability and Function of Encapsulated Porcine Islets. Adv. Healthc. Mater..

[96] Jha A., Moore E., Yigsr A. (2024). Laminin-derived peptide, dictates a concentration-dependent impact on macrophage phenotype response. Cell. Mol. Bioeng..

[97] Enck K., Tamburrini R., Deborah C., Gazia C., Jost A., Khalil F., Alwan A., Orlando G., Opara E.C. (2021). Effect of alginate matrix engineered to mimic the pancreatic microenvironment on encapsulated islet function. Biotechnol. Bioeng..

[98] Nicolas J., Magli S., Rabbachin L., Sampaolesi S., Nicotra F., Russo L. (2020). 3D extracellular matrix mimics: Fundamental concepts and role of materials chemistry to influence stem cell fate. Biomacromolecules.

[99] Farjaminejad S., Farjaminejad R., Hasani M., Garcia-Godoy F., Abdouss M., Marya A., Harsoputranto A., Jamilian A. (2024). Advances and Challenges in Polymer-Based Scaffolds for Bone Tissue Engineering: A Path Towards Personalized Regenerative Medicine. Polymers.

[100] Ghasemi A., Akbari E., Imani R. (2021). An overview of engineered hydrogel-based biomaterials for improved β-cell survival and insulin secretion. Front. Bioeng. Biotechnol..

[101] Paredes J.G.A., Spasojevic M., Faas M.M., de Vos P. (2014). Immunological and technical considerations in application of alginate-based microencapsulation systems. Front. Bioeng. Biotechnol..

[102] Lukova P., Kokova V., Baldzhieva A., Murdjeva M., Katsarov P., Delattre C., Apostolova E. (2024). Alginate from *Ericaria crinita* Possesses Antioxidant Activity and Attenuates Systemic Inflammation via Downregulation of Pro-Inflammatory Cytokines. Mar. Drugs.

[103] Torres M.L., Fernandez J.M., Dellatorre F.G., Cortizo A.M., Oberti T.G. (2019). Purification of alginate improves its biocompatibility and eliminates cytotoxicity in matrix for bone tissue engineering. Algal Res..

[104] Valente J., Valente T., Alves P., Ferreira P., Silva A., Correia I.J. (2012). Alginate-based scaffolds for bone tissue engineering. Mater. Sci. Eng. C.

[105] Eskandani M., Derakhshankhah H., Jahanban-Esfahlan R., Jaymand M. (2023). Biomimetic alginate-based electroconductive nanofibrous scaffolds for bone tissue engineering application. Int. J. Biol. Macromol..

[106] Beltran-Vargas N.E., Peña-Mercado E., Sánchez-Gómez C., Garcia-Lorenzana M., Ruiz J.C., Arroyo-Maya I. (2022). Sodium alginate/chitosan scaffolds for cardiac tissue engineering: The influence of its three-dimensional material preparation and the use of gold nanoparticles. Polymers.

[107] Baheiraei N., Razavi M., Ghahremanzadeh R. (2023). Reduced graphene oxide coated alginate scaffolds: Potential for cardiac patch application. Biomater. Res..

[108] Rioux Y., Fradette J., Maciel Y., Bégin-Drolet A., Ruel J. (2022). Biofabrication of sodium alginate hydrogel scaffolds for heart valve tissue engineering. Int. J. Mol. Sci..

[109] Dienes H.P., Drebber U. (2010). Pathology of immune-mediated liver injury. Dig. Dis..

[110] Bangru S., Pedersen M., Singal A., Mufti A. (2018). Survey of liver transplantation practices for severe acute alcoholic hepatitis. Liver Transplant..

[111] Rajalekshmi R., Shaji A.K., Joseph R., Bhatt A. (2021). Scaffold for liver tissue engineering: Exploring the potential of fibrin incorporated alginate dialdehyde-gelatin hydrogel. Int. J. Biol. Macromol..

[112] Jeon H., Kang K., Park S.A., Kim W.D., Paik S.S., Lee S.H., Jeong J., Choi D. (2017). Generation of multilayered 3D structures of HepG2 cells using a bio-printing technique. Gut Liver.

[113] Boontheekul T., Kong K.J., Mooney D.J. (2005). Controlling alginate gel degradation utilizing partial oxidation and bimodal molecular weight distribution. Biomaterials.

[114] Chen M., Tian D., Zhang J., Wang Q., Wang X., Yu X., Zhang C.W. (2012). Preparation and characterization of oxidized alginate covalently cross-linked galactosylated chitosan scaffold for liver tissue engineering. Mater. Sci. Eng. C.

[115] Mann D.L., Lee R.J., Coats A.J.S., Neagoe G., Dragomir D., Pusineri E., Piredda M., Bettari L., Kirwan B.A., Dowling R. (2016). One-year follow-up results from AUGMENT-HF: A multicentre randomized controlled clinical trial of the efficacy of left ventricular augmentation with Algisyl in the treatment of heart failure: One-year follow-up results from AUGMENT-HF. Eur. J. Heart Fail..

[116] Syanda A.M., Kringstad V.I., Blackford S.J.I., Kjesbu J.S., Ng S.S., Ma L., Xiao F., Coron A.E., Rokstad A.M.A., Modi S. (2022). Sulfated Alginate Reduces Pericapsular Fibrotic Overgrowth on Encapsulated cGMP-Compliant hPSC-Hepatocytes in Mice. Front. Bioeng. Biotechnol..

[117] Strand B.L., Coron A.E., Skjåk-Bræk G. (2017). Current and future perspectives on alginate encapsulated pancreatic islet. Stem Cells Transl. Med..

[118] Keymeulen B., De Groot K., Jacobs-Tulleneers-Thevissen D., Thompson D.M., Bellin M.D., Kroon E.J., Daniels M., Wang R., Jaiman M., Kieffer T.J. (2024). Encapsulated stem cell–derived β cells exert glucose control in patients with type 1 diabetes. Nat. Biotechnol..

[119] Corona B.T., Ward C.L., Baker H.B., Walters T.J., Christ G.J. (2014). Implantation of in vitro tissue engineered muscle repair constructs and bladder acellular matrices partially restore in vivo skeletal muscle function in a rat model of volumetric muscle loss injury. Tissue Eng. Part A.

[120] Thierry B., Charles H., Wassmer F.L., Kevin B., Laura M.F., Juliette B.R., Hanna A.P., Ekaterine B. (2022). From islet of Langerhans transplantation to the bioartificial pancreas. La. Presse Médicale.

[121] Papas K.K., De Leon H., Suszynski T.M., Johnson R.C. (2019). Oxygenation strategies for encapsulated islet and beta cell transplants. Adv. Drug Deliv. Rev..

[122] Kakaei N., Izadi Z., Mohammadi G., Ahmadi A., Amirian R., Moloudi M.R. (2024). A thin disc-shaped macrocapsule for transplantation of oxygen carrier-laden alginate hydrogel-encapsulated pancreatic islets in diabetic mice. Mater. Adv..

[123] Stratakis E. (2018). Novel biomaterials for tissue engineering. Int. J. Mol. Sci..

[124] Trucillo P. (2024). Biomaterials for Drug Delivery and Human Applications. Materials.

[125] Matsumoto S., Abalovich A., Wechsler C., Wynyard S., Elliott R.B. (2016). Clinical benefit of islet xenotransplantation for the treatment of type 1 diabetes. eBioMedicine.

[126] Yang X., Lu Z., Wu H., Li W., Zheng L., Zhao J. (2018). Collagen-alginate as bioink for three-dimensional (3D) cell printing based cartilage tissue engineering. Mater. Sci. Eng. C Mater. Biol. Appl..

[127] Kosik-Kozioł A., Costantini M., Bolek T., Szöke K., Barbetta A., Brinchmann J., Święszkowski W. (2017). PLA short sub-micron fiber reinforcement of 3D bioprinted alginate constructs for cartilage regeneration. Biofabrication.

[128] Nguyen D., Hägg D.A., Forsman A., Ekholm J., Nimkingratana P., Brantsing C., Kalogeropoulos T., Zaunz S., Concaro S., Brittberg M. (2017). Cartilage tissue engineering by the 3D bioprinting of iPS cells in a nanocellulose/alginate bioink. Sci. Rep..

[129] Chu Y., Huang L., Hao W., Zhao T., Zhao H., Yang W., Xie X., Qian L., Chen Y., Dai J. (2021). Long-term stability, high strength, and 3D printable alginate hydrogel for cartilage tissue engineering application. BioMed Mater..

[130] Purcell E.K., Singh A., Kipke D.R. (2009). Alginate composition effects on a neural stem cell-seeded scaffold. Tissue Eng. Part C Methods.

[131] Wei L., Tan J., Li L., Wang H., Liu S., Chen J., Weng Y., Liu T. (2022). Chitosan/Alginate Hydrogel Dressing Loaded FGF/VE-Cadherin to Accelerate Full-Thickness Skin Regeneration and More Normal Skin Repairs. Int. J. Mol. Sci..

[132] Egorikhina M.N., Timofeeva L.B., Rubtsova Y.P. (2025). Study of the Effectiveness of Skin Restoration Using a Biopolymer Hydrogel Scaffold with Encapsulated Mesenchymal Stem Cells. Int. J. Mol. Sci..

[133] Maryam G.N., Naeimeh A.G., Azizeh R.D.B. (2023). Synergistic effect of chitosan-alginate composite hydrogel enriched with ascorbic acid and alpha-tocopherol under hypoxic conditions on the behavior of mesenchymal stem cells for wound healing. Stem Cell Res. Ther..

[134] Uppuluri V.N.V.A., Sathanantham S.T., Elumalai L. (2022). Polymeric Hydrogel Scaffolds: Skin Tissue Engineering and Regeneration. Adv. Pharm. Bull..

[135] Niu C., Wang L., Ji D. (2022). Fabrication of SA/Gel/C scaffold with 3D bioprinting to generate micro-nano porosity structure for skin wound healing: A detailed animal in vivo study. Cell Regen..

[136] Wang H., Chen X., Wen Y., Li D., Sun X., Liu Z., Yan H., Lin Q. (2022). A Study on the Correlation between the Oxidation Degree of Oxidized Sodium Alginate on Its Degradability and Gelation. Polymers.

[137] Rahman M.M. (2024). Sources, extraction, and applications of alginate: A review. SN Appl. Sci..

[138] Reakasame S., Boccaccini A.R. (2018). Oxidized alginate-based hydrogels for tissue engineering applications: A review. Biomacromolecules.

[139] Gajić S., Savić I.M., Svirčev I.M. (2023). Preparation and characterization of alginate hydrogels with high water-retaining capacity. Polymers.

[140] Li G., Marican M.H.M., Gan S.W., Li T., Zhang L., Liu J. (2024). Replicating Mg scaffold via 3D printing sacrificial template. J. Manuf. Process..

